# Role of CEBPa in trophectoderm competence installment

**DOI:** 10.1126/sciadv.ady1693

**Published:** 2025-11-28

**Authors:** Xiao Wei, Irepan Salvador-Martinez, Maciej Meglicki, Marcos Plana-Carmona, Antonios Klonizakis, Barbara Pernaute, Manuel Irimia, Gregoire Stik, Mina Popovic, Guillem Torcal Garcia, Holger Heyn, Magdalena Zernicka-Goetz, Thomas Graf

**Affiliations:** ^1^ Centre for Genomic Regulation (CRG), The Barcelona Institute of Science and Technology, Dr. Aiguader 88, Barcelona 08003, Spain.; ^2^ Single Cell Genomics, Centro Nacional de Análisis Genómico, Barcelona 08028, Spain.; ^3^ Mammalian Embryo and Stem Cell Group, Department of Physiology, Development and Neuroscience, University of Cambridge, Downing Street, Cambridge CB2 3EG, UK.; ^4^ Department of Medicine and Life Sciences, Universitat Pompeu Fabra (UPF), Barcelona 08005, Spain.; ^5^ ICREA, Pg. Lluis Companys 23, Barcelona 08010, Spain.; ^6^ Research and Development, Eugin Group, Barcelona 08006, Spain.; ^7^ Universitat de Barcelona (UB), Barcelona 08007, Spain.; ^8^ Division of Biology and Biological Engineering, California Institute of Technology, Pasadena, CA 91125, USA.

## Abstract

During mouse embryogenesis, totipotency is gradually lost, and, at the 16-cell stage, blastomeres begin to bifurcate into trophectoderm (future placenta) and inner cell mass (future fetus). Although this process is well studied, when and how blastomeres acquire the competence for lineage specification remains unclear. Here, we describe that CEBPa becomes up-regulated at the transition from the two- to the four-cell stage by NR5A2 and is also selectively expressed in the trophectoderm at the blastocyst stage. Its knockout decreases the proportion of trophectoderm cells and delays the morula to blastocyst transition. Conversely, CEBPa overexpression in mouse embryonic stem cells, used as a proxy, drives their differentiation into trophectoderm-like cells, enabling the identification of CEBPa-regulated trophectoderm-specific enhancers. A subset of these enhancers, associated with key trophectoderm-related transcription factor genes, is primed or activated in four- and eight-cell embryos. Together, our data suggest that CEBPa plays a role in the installment of trophectoderm competence before the first lineage bifurcation and in trophectoderm specification.

## Teaser

CEBPa is expressed in early mouse embryos and has a role in competence installment and specification of the trophectoderm lineage.

## INTRODUCTION

The transition from a pluripotent or totipotent state to lineage commitment is a cornerstone of developmental biology, yet the molecular basis of how cells become competent to differentiate remains incompletely understood. In 1940, Conrad Waddington introduced the term “developmental competence” to describe the capacity of multipotent progenitor cells to respond to specific inductive cues and give rise to defined cell fates: “The notion of competence…is tested by finding out what types of development a piece of tissue can…carry out” (*1*). Decades later, Ken Zaret provided a molecular framework for this concept by demonstrating that the transcription factor (TF) GATA4 could prime endodermal enhancers, paving the way for future TF binding and gene activation during differentiation (*2*, *3*). These and subsequent studies suggest that developmental competence involves a preparatory phase, marked not by overt gene expression changes but by enhancer priming, chromatin accessibility, and the coexpression of competing transcriptional programs that maintain bipotency (*4*–*9*). In this study, we use a CEBPa overexpression model in embryonic stem cells (ESCs) as a proxy to investigate the earliest stages of mouse embryogenesis to understand how developmental competence is first established and how it predisposes blastomeres toward lineage bifurcation.

Mammalian embryogenesis begins when the totipotent zygote divides into two blastomeres (*10*). At the two-cell stage, embryos undergo zygotic genome activation (ZGA), and, as cleavage divisions progress, totipotency is gradually lost (*11*–*14*). At the 16-cell stage, the outside cells form the trophectoderm (TE), and the inside cells generate the inner cell mass (ICM) that will become the epiblast (which gives rise to the lineages of the future fetus) and primitive endoderm (*15*–*18*). When the blastocyst implants, the TE establishes the extraembryonic ectoderm, ultimately the placenta, and mediates implantation into the uterus (*19*, *20*).

It was long believed that all blastomeres in the embryo were identical and totipotent until the 16-cell stage. However, findings over the past two decades have challenged this view. Studies in both mouse and human embryos (*21*–*30*) have revealed that blastomeres begin to diverge as early as the two-cell stage and that these early differences can bias subsequent cell fate. For example, lineage tracing and time-lapse imaging of mouse embryos have shown that one of the two blastomeres at the two-cell stage tends to contribute preferentially to the epiblast and polar TE, while the other is biased toward the primitive endoderm and mural TE (*31*–*33*). In contrast, a separate study using light-sheet microscopy reported that significant lineage bias toward either the TE or ICM emerges only at the 16-cell stage (*34*). Despite these insights, the mechanisms that initiate the TE-ICM bifurcation remain poorly understood.

A number of TFs have been shown to act early and to be involved in the specification of TE and ICM. The ICM regulators, KLF5 and NR5A2, and the TE regulators, TFAP2C and TEAD4, bind to and activate genes affiliated with both lineages in preimplantation embryos before lineage diversification (*8*, *9*, *35*, *36*). Moreover, overexpression of KLF5 in mouse ESCs can induce the formation of bipotential cells (*35*). Despite these findings, how blastomeres balance lineage priming with the maintenance of cell fate plasticity remains largely unknown.

An important event regulating the ICM-TE bifurcation is the inactivation of Hippo signaling in the outer cells at the 16-cell stage, leading to the nuclear translocation of YAP. Nuclear YAP forms a complex with TEAD4 to induce zygotic *Cdx2* expression (*14*, *37*–*41*). CDX2, in turn, up-regulates TE-related genes such as *Id2*, *Eomes*, and *Elf5*, acting in parallel with GATA3 (*42*–*44*). In contrast, elevated Hippo signaling in the ICM inactivates YAP, resulting in the up-regulation of ICM-specific genes (*14*, *45*, *46*). In addition, overexpressing CDX2, GATA3, EOMES, TFAP2C, TEAD4, or ELF5 in ESCs, which are derived from the ICM, can at least partially activate the TE program (*42*, *47*, *48*). These factors thus form the core TE-associated TFs.

The TF CEBPa (CCAAT/enhancer-binding protein alpha) regulates the differentiation of a variety of cell types, including myeloid cells, adipocytes, and hepatocytes (*49*–*53*). In the hematopoietic system, it drives the formation of granulocyte/macrophage progenitors, with knockout (KO) mice exhibiting an early differentiation block and dying perinatally (*51*, *54*). CEBPa is also expressed in the TE, where its germline KO delays blastocyst formation in vitro and down-regulates *Il6* expression (*55*). Moreover, simultaneous deletion of *Cebpa* and its homolog *Cebpb* results in a placental defect, causing embryonic death around embryonic day 10 (E10) (*56*). CEBPa is a potent inducer of B cell–to–macrophage transdifferentiation (*57*–*59*), acting as a pioneer factor that collaborates with endogenous PU.1 (*60*). Last, a pulse of CEBPa in B cells before activation of the Yamanaka factors markedly enhances the cells’ reprogramming efficiency into induced pluripotent stem cells (*61*).

Here, we show that CEBPa expression is initiated in uncommitted four-cell stage blastomeres and, later, is restricted to the TE layer of blastocysts. Ectopic expression of CEBPa in ESCs drives differentiation toward TE-like cells (TELCs) by priming and activating enhancers of key TE-associated TFs, resulting in the up-regulation of TE genes and TE fate acquisition. Notably, in early blastomeres, a subset of these CEBPa-regulated enhancers is already activated or primed within the body of TE-associated TFs, establishing a transcriptional landscape permissive for TE specification. Together, these findings position CEBPa as a very early regulator of TE competence, linking transcriptional priming in totipotent blastomeres to the first lineage bifurcation in the mammalian embryo.

## RESULTS

### CEBPa becomes heterogeneously expressed at the two- to four-cell transition and in the TE lineage

**Fig. 1.**
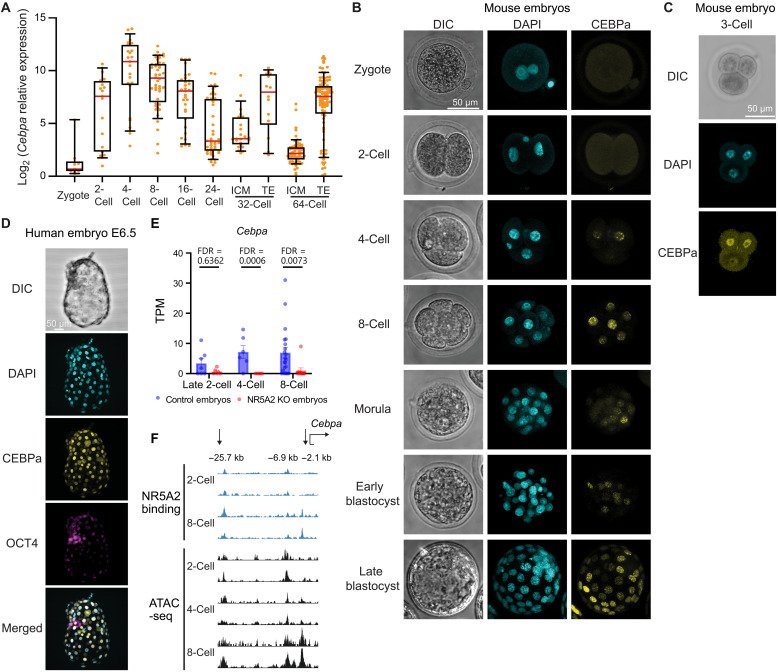
CEBPa is heterogeneously up-regulated by NR5A2 in four- and eight-cell embryos and selectively expressed in the TE layer. (**A**) Expression of *Cebpa* normalized to *Gapdh* (RT-qPCR) in mouse preimplantation embryos (*62*). Boxplots and whiskers depict 10 to 90 percentiles with the red line indicating median values. TE, trophectoderm; ICM, inner cell mass. (**B**) Mouse embryos at different developmental stages viewed by differential interference contrast (DIC) microscopy and immunofluorescence (IF) for DNA [4′,6-diamidino-2-phenylindole (DAPI)] and CEBPa expression. (**C**) Expression of CEBPa in a three-cell embryo. (**D**) Human embryo, grade 6AA, at day 6.5, viewed by DIC and IF for DAPI, CEBPa, and OCT4 expression. (**E**) Effect of zygotic NR5A2 KO on *Cebpa* expression at late two-cell, four-cell, and eight-cell stages analyzed from public RNA-seq data (*66*). (**F**) Blue lanes: duplicates of NR5A2 binding at *Cebpa* upstream regions in two-cell and eight-cell embryos visualized by public CUT&RUN data (*8*); Black lanes: duplicates of chromatin accessibility in two- to eight-cell stage embryos visualized by published ATAC-seq data (*36*). NR5A2-bound regions likely to regulate *Cebpa* expression are indicated by black arrows.

Published single-cell reverse transcription quantitative polymerase chain reaction (RT-qPCR) (*62*) and single-cell RNA sequencing (scRNA-seq) data (*27*) reveal that *Cebpa* expression is initiated at the two-cell stage in mouse embryos, peaks at the four-cell stage, and shows a second peak in TE cells at the blastocyst stage (Fig. 1A and fig. S1A). Our immunofluorescence analysis confirms that CEBPa is undetectable in zygotes and weakly expressed in some two-cell blastomeres but robustly expressed in four- and eight-cell embryos (Fig. 1B and fig. S1B). Notably, in four-cell embryos, CEBPa levels vary among blastomeres and positively correlate with expression of the TE-associated TF TEAD4 (fig. S1, C and D), suggesting that this heterogeneity may be functionally relevant. In mitotic cells, CEBPa localizes to pericentromeric regions of chromosomes (fig. S1E).

To determine the timing of CEBPa up-regulation, we examined three-cell stage embryos. We found that the two smaller, newly divided blastomeres exhibited a stronger CEBPa signal (Fig. 1C), indicating that CEBPa becomes up-regulated during the two- to four-cell stage transition. After the eight-cell stage, CEBPa expression decreases, but it is reactivated in a subset of TE cells at the blastocyst stage (Fig. 1, A and B; and fig. S1, F and G). In human embryos, *CEBPA* is detected later, at the morula to the blastocyst transition (fig. S1H) (*63*) and is largely restricted to the TE of late blastocysts (Fig. 1D). Together, these findings show that, in mouse embryogenesis, CEBPa becomes heterogeneously expressed during the two- to four-cell transition and in a subset of TE cells at the blastocyst stage.

### NR5A2 regulates *Cebpa* expression

Because the TFs DUX and NR5A2 are up-regulated during ZGA and highly expressed in two-cell embryos (*13*, *64*–*68*), we explored whether *Cebpa* is a target of these regulators. Analyzing published RNA sequencing (RNA-seq) data (*69*) revealed that forced expression of DUX in mouse ESCs (fig. S2A), which induces the formation of two-cell–like cells (*69*), caused a marked *Cebpa* up-regulation (73-fold), while seven key TE-TFs were up-regulated <2-fold (fig. S2, B and C). The same dataset revealed that DUX binds to a −7.1-kb candidate enhancer element of *Cebpa*, whose chromatin accessibility increases after DUX overexpression (fig. S2D). As determined by Assay for Transposase-Accessible Chromatin using sequencing (ATAC-seq) (*36*, *70*), this region is also transposase-accessible in two-cell to eight-cell stage embryos, consistent with the possibility that DUX is a potential activator of CEBPa.

Analysis of a published dataset describing NR5A2 as a regulator of *Tead4* and *Tfap2c* expression (*66*) revealed that a NR5A2 KO caused the silencing of *Cebpa* expression in four- and eight-cell embryos, comparable to what was reported for *Tead4* (Fig. 1E and fig. S2E). Moreover, integrating published datasets revealed three potential enhancer elements of *Cebpa* (−25.7, −6.9, and −2.1 kb) bound by NR5A2 (*8*) and also exhibit accessible chromatin in four- to eight-cell embryos (Fig. 1F) (*36*). Of these, the −25.7- and the −2.1-kb sites showed reduced accessibility upon NR5A2 depletion in eight-cell embryos (fig. S2F) (*66*), in line with their function as NR5A2-regulated enhancers.

Collectively, these findings indicate that NR5A2 regulates the expression of *Cebpa* during the two- to four-cell transition. They also suggest a similar role for DUX, although its physiological significance still needs to be fully established.

### CEBPa contributes to the timely blastocyst formation and TE development

**Fig. 2.**
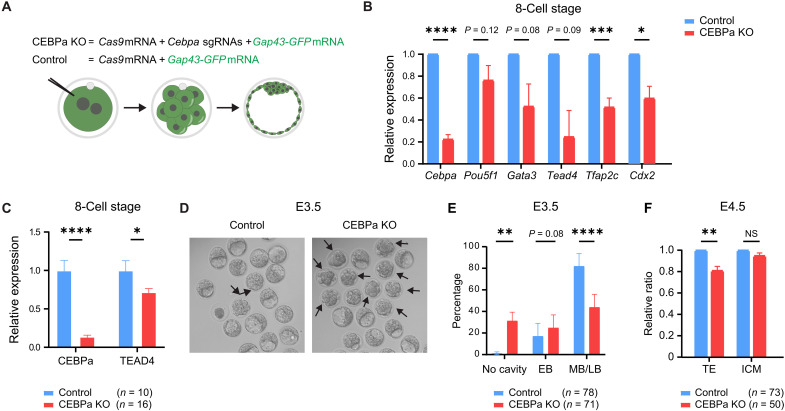
CEBPa contributes to the timely blastocyst formation and TE development. (**A**) Strategy to knock out CEBPa by CRISPR-Cas9 in zygotes. *Gap43-GFP* was used as a reporter for injected *Cas9* mRNA and 2 gRNAs specific for *Cebpa*. (**B**) Relative gene expression of lineage markers, as determined by RT-qPCR in CEBPa KO eight-cell embryos normalized to controls (means ± SEM). Statistical significance was determined using the *t* test. *P ≤ 0.05; ***P ≤ 0.0005; ****P ≤ 0.00005. (**C**) Relative expression of TEAD4 in late eight-cell embryos comparing wild type with CEBPa KO embryos measured by IF (shown as means ± SEM). (**D**) Representative images of control and CEBPa KO E3.5 embryos, with developmentally delayed embryos indicated by black arrows. (**E**) Quantification of cavitation rates of embryos from (D), shown as means ± SD. **P ≤ 0.005. EB, early blastocyst with <1/2 cavity volume of the embryo; MB, middle blastocyst with >1/2 volume of the embryo; LB, late blastocyst with the cavity filling the embryo and ICM on the side. (**F**) Relative numbers of TE and ICM cells in E4.5 CEBPa KO embryos normalized to controls (means ± SD). Statistical significance determined as in (G). NS, not significant.

To investigate the role of CEBPa in embryonic development, we used CRISPR-Cas9–mediated gene editing to generate *Cebpa* KO embryos. Zygotic nuclei were microinjected with a combination of *Cebpa*-specific guide RNAs, *Cas9* mRNA, and *Gap43-GFP* mRNA as a tracer (Fig. 2A). This approach achieved 90% KO efficiency, with 28 of the 32 embryos showing marked reduced expression of CEBPa at the blastocyst stage (fig. S2G).

Loss of CEBPa led to a significant down-regulation of genes encoding key TE-associated TFs: *Tfap2c* and *Cdx2* expression was markedly reduced, while *Tead4* and *Gata3* mRNA levels were also diminished in eight-cell embryos. In contrast, the expression of the pluripotency/ICM marker *Pou5f1* (encoding OCT4) remained unaffected (Fig. 2B). These transcriptional changes were mirrored at the protein level, with zygotic *Cebpa*-deficient late eight-cell embryos showing significantly reduced TEAD4 expression compared to controls (Fig. 2C).

Functionally, CEBPa ablation delayed the transition from morula to blastocyst. At E3.5, only 44% of KO embryos had formed mid or late blastocysts, compared to 82% in controls (Fig. 2, D and E). Further analysis at E4.5 revealed a ~20% reduction in the proportion of CDX2-positive TE cells in *Cebpa* KO embryos, while the proportion of ICM cells remained unchanged (Fig. 2F). Notably, this decrease is substantial given that only a subset of TE cells normally expresses CEBPa. Together, these findings indicate that CEBPa promotes the expression of multiple TE-associated TFs, supports timely blastocyst formation, and contributes to the specification and expansion of the TE lineage.

### Ectopic expression of CEBPa in ESCs induces the formation of TE lineage cells

**Fig. 3.**
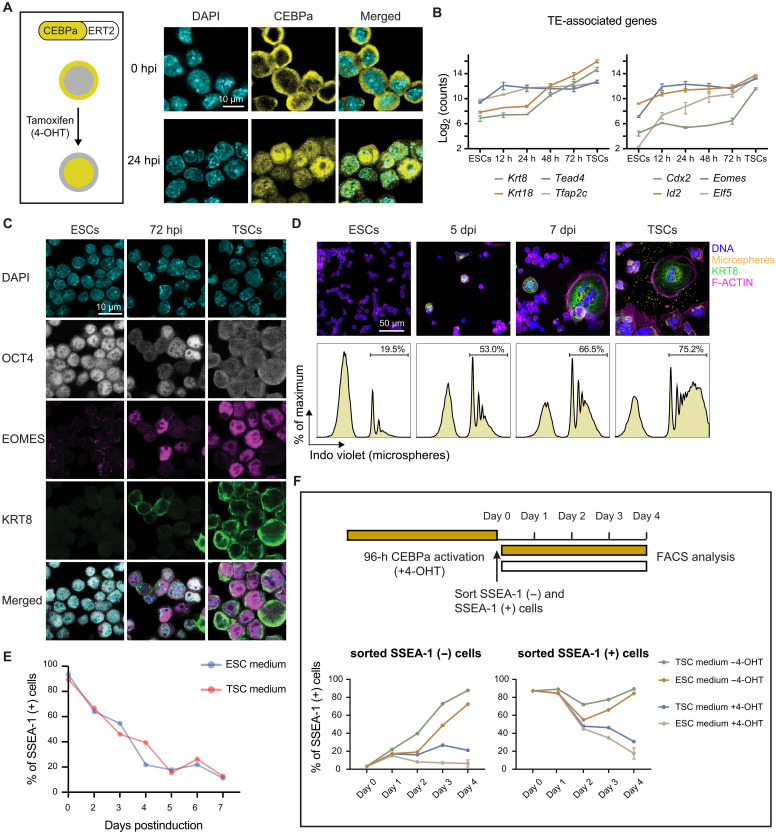
Ectopic expression of CEBPa in ESCs induces the formation of TELCs. (**A**) Left: Schematics of CEBPa activation through nuclear translocation induced upon tamoxifen (4-OHT) treatment of an ESC cell clone expressing CEBPa-ERT2-tdTomato. Yellow areas represent the localization of CEBPa before and after induction, as also shown on the right with actual cells at 0 and 24 hours postinduction (hpi). (**B**) Expression of TE-associated genes after CEBPa induction. Data are represented as means ± SEM among biological replicates. h, hours. (**C**) Immunostaining for OCT4, EOMES, and KRT8 in 72-hpi CEBPa-ER cells, as well as in ESCs and TSCs as controls. (**D**) Phagocytic capacity of uninduced CEBPa-ER cells, as well as of 5- and 7-day–induced cells, determined by incubation with fluorescent carboxylated microspheres, with TSCs as control. Top: IF images of cells stained for DNA (picogreen), F-ACTIN (phalloidin), and KRT8. Bottom: Fluorescence-activated cell sorting (FACS)–derived histograms depicting percentages of internalized microspheres. dpi, days postinduction. (**E**) Kinetics of the cell surface markers SSEA-1 (indicative of pluripotency) measured by FACS of CEBPa-induced cells cultured in ESC and TSC media. (**F**) Top: Experimental design. CEBPa-ER cells were treated with 4-OHT for 96 hours, sorted for SSEA-1 low and high cells, and replated in the absence or presence of the inducer. Bottom: Kinetics of SSEA-1 expression for both fractions after replating. Data represent means ± SEM of two replicates.

On the basis of these observations, we hypothesized that transient CEBPa expression may endow ESCs with TE competence, mimicking the four-cell stage blastomeres, whereas prolonged CEBPa expression may drive terminal TE differentiation. To test this and to be able to dissect the temporal effects of CEBPa, we generated mouse ESCs that stably express an inducible form of CEBPa (CEBPa-ERT2-tdTomato; CEBPa-ER in brief) (Fig. 3A). Treatment of these cells with 4-hydroxy tamoxifen (4-OHT) translocates CEBPa from the cytoplasm to the nucleus (Fig. 3A), leading to the factor’s activation. Such an induction generated the formation of flat colonies resembling TE stem cells (TSCs) (fig. S3A). RNA-seq with cells induced for 0, 12, 24, 48, and 72 hours showed a robust up-regulation of the key TE-associated TF genes *Gata3*, *Tead4*, *Tfap2c*, *Eomes*, *Elf5*, and *Id2* and a weaker activation of *Cdx2* (Fig. 3B and fig. S3B). In contrast, genes associated with pancreatic, cardiac, and neural tissues remained unchanged (fig. S3C). Immunofluorescence of cells at 72 hours postinduction (hpi) revealed expression of the TE-restricted markers EOMES and KRT8 in a subset of cells, with a concomitant reduction of the key pluripotency regulator OCT4 (Fig. 3C) (*71*, *72*). After prolonged induction, many cells acquired a morphology reminiscent of trophoblast giant cells (Fig. 3D), known to be phagocytic (*20*, *73*). We therefore examined the phagocytic capacity of CEBPa-induced cells by the uptake of carboxylated microspheres. Five- and 7-day–induced cultures result in large cells with numerous internalized microspheres comparable to TSC (Fig. 3D).

To compare the differentiation efficiency of CEBPa-ER in ESC and TSC media, we measured the expression of the ESC-restricted cell surface marker stage-specific embryonic antigen-1 (SSEA-1) (*74*) and the TE-associated marker stem cell antigen-1 (SCA-1) (*75*) by flow cytometry [fluorescence-activated cell sorting (FACS)] at different times after induction. This showed similar kinetics of SSEA-1 down-regulation and SCA-1 up-regulation under both conditions, while parental E14 ESCs treated with 4-OHT showed no response (Fig. 3E and fig. S3, D to G). Expression of core TE genes (*Krt8*, *Gata3*, *Id2*, and *Elf5*) assessed by RT-qPCR showed similar degrees of activation, with the exception of *Eomes* that showed a stronger up-regulation in TSC medium (fig. S3H). In addition, markers of trophoblast giant cells (*Pl1*, *Plf*, and *Hand1*), syncytiotrophoblasts (*Gcm1*), and glycogen trophoblast cells (*Pcdh12*) were similarly up-regulated upon culture in TSC medium (fig. S3I). As measured by the signal of CellTrace Violet–stained CEBPa-ER cells over time, CEBPa induction led to a slowdown of cell division (fig. S3J). To exclude the possibility that the differentiation toward TE cells resulted from the CEBPa-induced cell cycle arrest, we tested the effect of the MYC inhibitor 10058-F4, known to be required to maintain the proliferation potential of ESCs (*76*). We observed that the addition of 100 μM 10058-F4 induced a comparable proliferation arrest as after CEBPa induction, yet, in this case, the cells remained SSEA-1 positive, suggesting that no TE differentiation was induced (fig. S3, J and K).

In summary, our results show that ectopic expression of CEBPa in ESCs induces the formation of TELCs at similar efficiencies in ESC and TSC culture media, indicating that CEBPa is able to override ESC culture conditions to specifically instate a TE identity. Prolonged CEBPa expression induced quiescence and the formation of mature TE cells, as indicated by their morphology, expression of late TE cell markers, and phagocytic capacity.

### Early induced TELCs are highly plastic

To determine whether early CEBPa-induced ESCs commit to the TE fate, we treated CEBPa-ER cells for 96 hours with 4-OHT, after which ~80% of the cells became SSEA-1 negative. We then sorted the negative cells and replated them with or without the inducer under both ESC and TSC culture conditions. Within 1 day after 4-OHT withdrawal, the cells reexpressed SSEA-1, reaching ESC levels within 4 days in both culture conditions, while they continued to differentiate into TELCs in the presence of 4-OHT (Fig. 3F). Sorted SSEA-1–positive cells used as a control showed a partial down-regulation of SSEA-1 2 days after withdrawal, which fully recovered after 4 days. In the presence of 4-OHT, SSEA-1–positive cells down-regulated SSEA-1 with similar kinetics as uninduced cells (Fig. 3, E and F).

These data show that TELCs obtained after a 4-day activation of CEBPa in ESCs are highly plastic and model the bipotency state of uncommitted blastomeres. Our results also indicate that a limited exposure to CEBPa generates a competent state while prolonged exposure drives full-fledged TE differentiation (fig. S3L).

### Single-cell analyses reveal progressive ESC to TE-lineage transitions and the stepwise activation of TE-associated genes

**Fig. 4.**
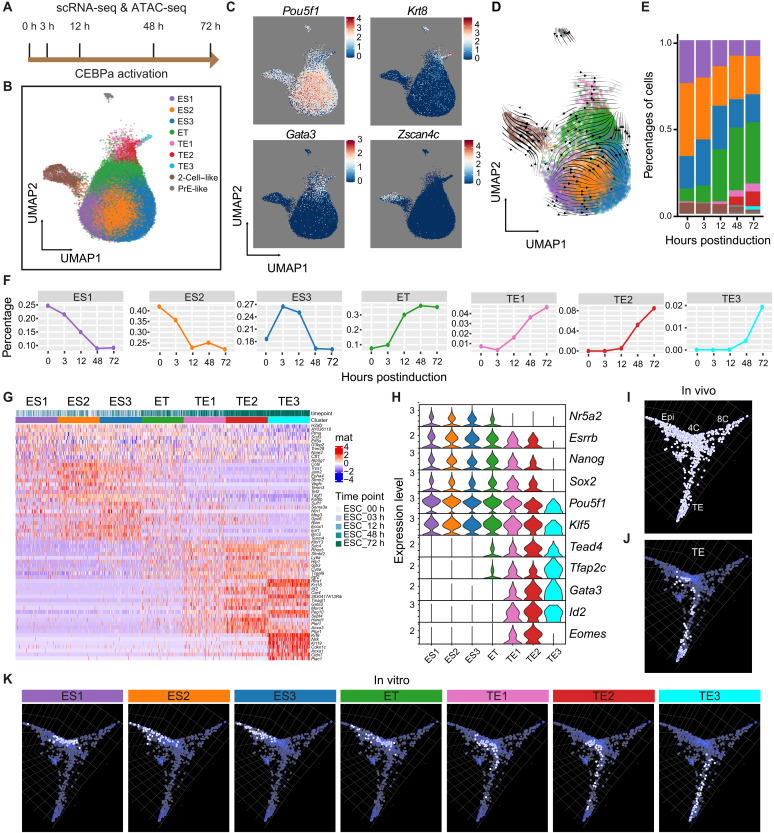
Single-cell analyses enable the temporal resolution of CEBPa-induced TE lineage formation and visualize the branching. (**A**) Schematic of CEBPa-induced ESCs, sampled at different times for multiomics analysis (scRNA-seq and scATAC-seq). h, hours. (**B**) Uniform manifold approximation and projection (UMAP) of all the induced samples after Harmony integration and cell clustering based on scRNA-seq. Seven ESC- and TE-related clusters could be distinguished, as well as clusters of two-cell and primitive endoderm (PrE)–like cells. (**C**) Distribution of marker gene expression for pluripotency (*Pou5f1*), TE (*Krt8* and *Gata3*), and two-cell state (*Zscan4c*) in the UMAP. (**D**) RNA velocity analysis with scVelo (*119*) of the 12-hpi sample. (**E**) Stacked bar plots showing the proportion of cells within the different clusters at different time points. (**F**) Percentages of cells within each cluster at different time points. (**G**) Expression heatmap of the top 10 differentially expressed genes (DEGs) of each cluster, using 150 randomly sampled cells for each cluster. Gene expression was normalized and scaled relative to the entire dataset (mean = 0; SD = 1). The clusters are indicated with the color code shown in (B). (**H**) Violin plots of ESC- and TE-TF gene expression of the integrated samples, with cluster color codes as in (B). (**I**) Single-cell distribution, based on gene expression, within the 3D developmental landscape of induced cell clusters integrated with early embryo cell states (*11*) as determined by CeLaVi (*79*). The figure depicts an aggregate of 50 (or less) randomly chosen cells for each CEBPa-induced cell cluster and chosen embryonic cell stage (see also fig. S4D). (**J**) Distribution of TE cells within the 3D cell landscape. (**K**) Distribution of cells from the seven clusters obtained after CEBPa induction within the 3D cell landscape.

To obtain insights into the cell conversion mechanism, we conducted scRNA-seq and ATAC-seq multiomic analysis of cells induced up to 3 days, likely relevant for the earliest stages of embryo development. For this, CEBPa-ER ESCs were induced with 4-OHT for 0, 3, 12, 48, and 72 hours, and TSCs were used as controls (Fig. 4A). To analyze gene expression changes, different samples were integrated with Harmony (*77*) and highly variable genes (HVGs) subjected to dimensionality reduction and cell clustering analysis. We defined nine clusters that were depicted in a uniform manifold approximation and projection (UMAP): three ESC-related clusters (ES1, ES2, and ES3) enriched for the expression of *Pou5f1*, three TE-related clusters (TE1, TE2, and TE3) enriched for *Gata3*, and one cluster coexpressing similar levels of both markers, designated as the ET cluster (Fig. 4, B and C). In addition, we detected a *Gata6*-expressing primitive endoderm-like cluster and a *Zscan4*-expressing two-cell–like cluster (Fig. 4C and fig. S4A). Integrating data from E14 ESCs (*78*) in the UMAP and performing a velocity analysis showed that the ES1 cluster predominates in E14 ESCs and suggests that the subsequent clusters form in the order ES2, ES3, ET, TE1, TE2, and TE3 (Fig. 4D and fig. S4B). Of note, although all clusters were already detectable in uninduced CEBPa-ER cells, the proportion of cells in the TE-related clusters steadily increased over time while ESC-related clusters decreased (Fig. 4, E and F). In addition, we observed a gradual decrease of the two-cell–like cluster and an increase of the primitive endoderm-like cluster (fig. S4C), which were not studied further.

To delineate the ESC to TE conversion, we analyzed differentially expressed genes (DEGs) along the reprogramming trajectory, showing the stepwise up-regulation of TE-associated genes (Fig. 4G). Thus, *Tead4* and *Tfap2c* became expressed first in ET cells, followed by a burst of *Gata3* and *Id2* in TE1-TE3 cells, while *Eomes* was specifically expressed at the TE1 and TE2 stages. Conversely, ESC-affiliated regulators became silenced (at least partially), with *Nr5a2* being extinguished first at the TE1 stage followed by *Esrrb*, *Nanog*, and *Sox2* at the TE2 stage and *Pou5f1* and *Klf5* at the TE3 stage (Fig. 4H). Together, the induction of CEBPa in ESCs results in a progressive transition from ESC- to TE-related clusters via the stepwise up-regulation of TE-associated TF genes, silencing of ESC-related genes, and the formation of a distinct cell intermediate (ET).

### Visualizing the ESC to TE-lineage conversion within a 3D developmental landscape

To determine whether the TE clusters are transcriptionally similar to the TE in mouse embryos, we integrated single cells from seven stages of mouse preimplantation embryos (two-cell, four-cell, eight-cell, morula, ICM, TE, and epiblast) (*11*) with our 7 CEBPa-induced clusters. A three-dimensional (3D) developmental landscape with distinct four-cell, eight-cell, TE, and epiblast cell branches (Fig. 4, I and J, and fig. S4D) was generated based on single-cell gene expression data using a recently developed web-based tool (*79*). Within this landscape, ES1 cells localized to the upper part of the ICM region, while ES2 and ES3 cells extended toward the epiblast branch, followed by a progressive reorientation toward the TE branch. ET cells represent an intermediate stage that connects the ESC-TE branching (Fig. 4K). To quantify the similarity between our ESC-derived cell clusters and physiological states, we calculated the proportion of nearest neighbors. This demonstrated that the TE clusters are closely aligned with the physiological TE state and that the ES2 and ES3 clusters are closest to the epiblast state (fig. S4E). Thus, the induced cells appear to acquire an epiblast signature at the ES2 and ES3 stages before they revert to an ESC-TE intermediate (the ET stage) and then progressively differentiate into TELCs from the TE1 to the TE3 stage.

### CEBPa induces the priming and sequential activation of TE-associated enhancers

Lineage-restricted transcriptomes are determined by the binding of lineage-instructive TFs to specific sequence motifs within gene regulatory elements (GREs), including enhancers (*80*). Therefore, to investigate changes in chromatin accessibility induced by the binding of key lineage regulators, we analyzed the ATAC-seq data from our single-cell multiomics experiment. Cells from the ES clusters displayed open chromatin regions enriched for binding motifs of pluripotency factors such as OCT4 and SOX2. In contrast, the ET and TE clusters showed open chromatin regions enriched for binding motifs of TE-associated factors like TFAP2C and EOMES, alongside the closing of pluripotency-associated loci (fig. S5A). Consistent with the motif activity computed with chromVAR (*81*), expression of the corresponding pluripotency genes decreased, while TE-TF encoding genes increased (fig. S5A).

**Fig. 5.**
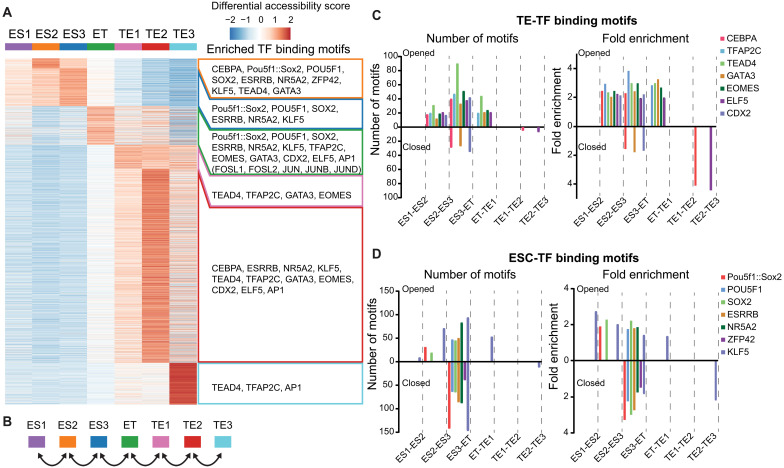
CEBPa induces the priming and sequential activation of TE-associated enhancers. (**A**) Heatmap showing the average (pseudo-bulk) accessibility of DARs for each cluster in the 72-hpi sample, with selected overrepresented motifs listed on the right (adjusted *P* value of <0.05). (**B**) Schematics showing pairwise comparisons of enriched TF binding motifs (adjusted *P* value of <0.05) within DARs across each transition. (**C**) Number of selected TE-TF motifs (left) and their fold enrichment (right) identified within each DAR that opens (top) or closes (bottom) in each transition. (**D**) Same analysis as in (C) but for selected ESC-TF motifs.

To assess differentially accessible regions (DARs) at each CEBPa-induced cell stage, we compared all stages to each other, revealing an enrichment of ESC-associated TF motifs in the DARs before the ET stage, while motifs for TE-associated TFs such as GATA3, CDX2, and EOMES were enriched after the ET stage onward (Fig. 5A). Similar findings were made by scoring selected TE- and ESC-TF motifs during the time course (fig. S5B). Notably, however, TEAD4 and GATA3 motifs already appeared at the ES2 stage, indicating an early opening of the corresponding putative TE-GREs. We also observed AP1 motifs enriched in the DARs from the ET stage onward, which were, however, not further studied. Of note, most of DARs at the ET cluster display chromatin accessibility values that fall in the middle of the dataset distribution (white areas), further supporting the notion that the ET cells represent an intermediate cell state between ESCs and TEs.

To explore which TF binding sites (representing putative regulatory sites) are opened or closed at each transition, we performed pairwise comparisons of chromatin accessibility changes, capturing major accessibility differences between transitions (Fig. 5B). The highest number of motifs that opened was found at the ES2-to-ES3 and ES3-to-ET transitions, while of closing was mostly seen at ES3 to ET (fig. S5C). The opening of TE-TF motifs was sequential, with CEBPA, TEAD4, TFAP2C, GATA3, EOMES, ELF5, and CDX2 motifs becoming accessible as early as at the ES2-to-ES3 transition, followed by additional sites with the same motifs opening at ES3 to ET and ET to TE1 (Fig. 5C and fig. S5D). The observation that TE-TF motifs already opened at ES2-ES3, i.e., before the relevant TE-associated TFs become expressed (fig. S5E), suggests that these sites are in a primed configuration. We also observed that CEBPA and ELF5 motif–enriched regions became less accessible in the TE2-to-TE3 transition, implying CEBPA as a pioneer factor that establishes initial TE identity, but dispensable for maintaining TE fate.

In contrast to TE-GREs, regions enriched for ESC-TF motifs (SOX2, POU5F1, ESRRB, NR5A2, and KLF5) became closed almost exclusively at the ES3-to-ET transition (Fig. 5D). However, unexpectedly, other sites with these motifs became opened during the same transition, suggesting that the corresponding TFs dynamically switch positions. Moreover, sites with POU5F1:SOX2, SOX2, and KLF5 motifs already became accessible during the ES1 to ES3 transitions (Fig. 5D and fig. S5F), raising the possibility that the corresponding factors collaborate with CEBPa in the early opening of these sites.

Together, our results indicate that CEBPa causes the opening of putative TE-GREs in a stepwise manner, with different subsets opening at early, intermediate, or late stages. A cohort of early opened sites was accessible even before TE-associated TF genes become expressed and is thus in a primed configuration. Conversely, sites with ESC-TF motifs became closed almost exclusively at the transition to ET cells, while other sites opened.

### CEBPa low and high levels instruct the formation of two distinct TELC fractions

**Fig. 6.**
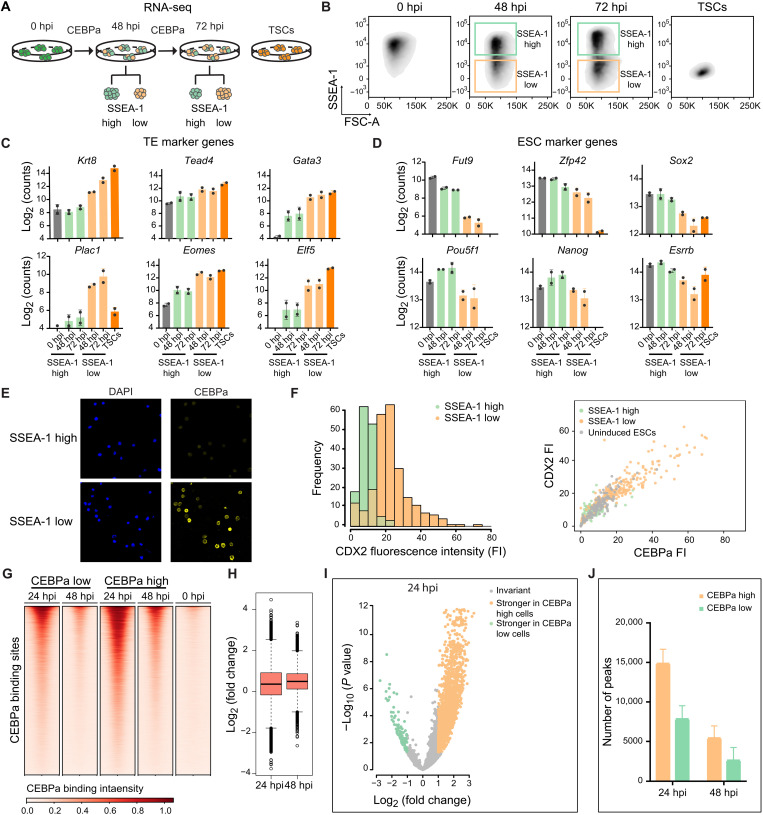
High and low levels of CEBPa drive the formation of two distinct TELC fractions. (**A**) Strategy to generate cell samples for RNA-seq analysis. CEBPa-ER cells were induced for 48 and 72 hours, and SSEA-1^high^ and SSEA-1^low^ fractions were sorted. Uninduced cells and TSCs were included as controls. Each condition consisted of two biological replicates. (**B**) FACS plots of cells induced for 48 and 72 hours, showing the sorting gates used to separate the two SSEA-1 fractions. (**C**) Average expression of TE-related genes, as determined by RNA-seq, of sorted SSEA-1^high^ and SSEA-1^low^ cell fractions at 48 and 72 hpi. Uninduced ESCs (0 hpi) and TSCs are shown as controls. Dots represent expression in two biological replicates. (**D**) As in (C), but for ESC-related genes. (**E**) Immunostaining for DAPI and CEBPa in SSEA-1^high^ and SSEA-1^low^ cells sorted at 72 hpi. (**F**) Left: Fluorescence intensity (FI) of CDX2 in SSEA-1^low^ (green bars) and SSEA-1^high^ cells (orange bars) at 72 hpi. Right: correlation of CEBPa with CDX2 expression levels in SSEA-1^low^ (green dots) and SSEA-1^high^ cells (orange dots). Gray dots correspond to uninduced cells. (**G**) CEBPa binding intensities ranked from top to bottom in CEBPa^low^ and CEBPa^high^ populations at 48 and 72 hpi, plus in 0-hpi cells. Genomic window shown corresponds to −0.3 and +0.3 kb from the center of the binding site. (**H**) Quantification of differences in CEBPa binding intensity between the CEBPa^high^ and CEBPa^low^ populations. Fold change: binding signal in CEBPa^high^ cells/CEBPa^low^ cells. (**I**) Comparison of CEBPa binding between CEBPa^low^ and CEBPa^high^ cells at 24 hpi. *P* values for fold change of binding signal (CEBPa^high^ cells/CEBPa^low^ cells) were calculated by DiffBind (*122*). (**J**) Numbers of CEBPa binding sites in CEBPa^high^ and CEBPa^low^ cells derived from 48- and 72-hpi cells identified by peak calling.

Single-cell analysis showed that induced cells at any given time point consist of various reprogramming stages, indicating heterogeneity of the ESC-TELC conversion. Supporting this, inspecting SSEA-1 expression of induced cells revealed the formation of distinct SSEA-1^low^ and SSEA-1^high^ cell fractions at intermediate time points, rather than showing a gradual and continuous down-regulation (fig. S6A). This suggests that the two SSEA-1 cell fractions convert into TELCs at different speeds. To examine their transcriptional profile, we sorted SSEA-1^low^ and SSEA-1^high^ cells at 48 and 72 hpi and performed RNA-seq (Fig. 6, A and B). As expected, SSEA-1^low^ cells exhibited more highly up-regulated TE-related genes (*Krt8*, *Tead4*, *Tfap2c*, *Gata3*, *Eomes*, *Elf5*, *Cdx2*, *Plet1*, and *Plac1*) and more strongly silenced ESC-associated TF genes (Fig. 6, C and D; and fig. S6, B and C), except for *Pou5f1* and *Nanog* that were slightly up-regulated in SSEA-1^high^ cells. Consistent with our interpretation, a principal components analysis (PCA) analysis showed that SSEA-1^low^ cells are closer to TSCs than SSEA-1^high^ cells (fig. S6D).

To test whether this heterogeneity is caused by different expression levels of CEBPa, we immunostained 72-hpi CEBPa-ER. This revealed stronger CEBPa signals in SSEA-1^low^ cells than those in SSEA-1^high^ cells (Fig. 6E). In addition, we observed a positive correlation between CEBPa and CDX2 as determined by immunostaining of 72-hpi cells (Fig. 6F). Together, our data suggest that SSEA-1^low^ cells contain higher levels of CEBPa and convert into TELCs more rapidly.

Next, we studied CEBPa binding in the different cell fractions using chromatin immunoprecipitation sequencing (ChIP-seq). Before this, we performed ChIP-seq on bulk 24-hpi CEBPa-ER cells. This yielded 30,507 peaks, while virtually none (208) were detected in uninduced cells. Of these, the CEBPa binding motif was ranked as the most enriched and centric motif and was detected in 55% of the sites (16,784 of 30,507) (fig. S6, E and F), proving the specificity and reliability of the ChIP-seq results. We then sorted SSEA-1^low^ and SSEA-1^high^ fractions (corresponding to CEBPa^high^ and CEBPa^low^ cells, respectively) from CEBPa-ER cells induced for 24 and 48 hours and again performed CEBPa ChIP-seq. Consistent with our previous data, CEBPa^high^ cells exhibited an overall stronger enhancer binding (1.3- to 1.4-fold) than CEBPa^low^ cells (Fig. 6, G and H). Moreover, of 9727 peaks detected at 24 hpi, 3203 showed >2-fold stronger binding in CEBPa^high^ cells, with only 113 the other way around, a skewing that was similarly observed with 48-hpi cells (Fig. 6I and fig. S6G). Last, we observed a 1.5- to 2-fold decrease in the number of CEBPa-bound sites from 24- to 48-hpi cell samples (Fig. 6J).

Collectively, our results indicate that, based on SSEA1 expression, CEBPa-induced cells can be separated into two distinct fractions differing by only 30 to 40% in CEBPa occupancy. The separation of SSEA-1 high and low fractions (corresponding to CEBPa^low^ and CEBPa^high^ cells) facilitated the identification of CEBPa-regulated chromatin regions, as described below.

### CEBPa drives chromatin accessibility changes through direct and indirect mechanisms

**Fig. 7.**
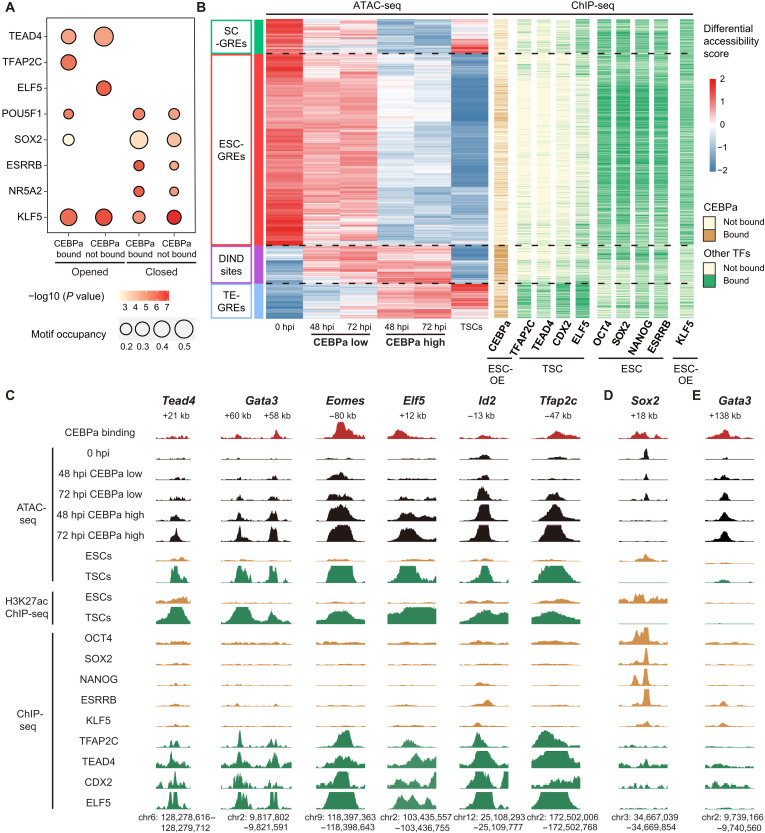
CEBPa overexpression enables the identification of TE- and ESC-associated GREs. (**A**) Bubble plot showing enrichment of key TE- and ESC-associated TF motifs in sites of CEBPa regulome bound or not bound by CEBPa. (**B**) Heatmap displaying chromatin accessibility of 9697 sites within CEBPa regulome in the conditions indicated at the bottom, averaged over duplicates. They were clustered into four groups of putative gene regulatory elements (GREs), as shown on the left. Sites bound by CEBPa at 24 hpi are indicated with brown lines, and other lineage-restricted TFs obtained from public ChIP-seq data with green lines (*35*, *82*–*85*). DIND sites, dose-independent sites. SC-GREs, stem cell-GREs. (**C** to **E**) Screenshots from the UCSC browser of individual TF enhancers and DIND site, showing CEBPa binding at 24 hpi (red peaks) and time-resolved chromatin accessibility changes in CEBPa^low^ and CEBPa^high^ cells (peaks in black). They also show chromatin accessibility, H3K27ac decoration (*85*, *86*), and binding of TE- and ESC-affiliated TFs in ESCs (brown peaks) and TSCs (green peaks) from published datasets (*35*, *82*–*85*). (C) TE-GREs, (D) ESC-GRE, and (E) DIND site. The browser gains for the respective tracks were maintained constant in all panels shown.

To explore changes of chromatin accessibility, we performed ATAC-seq experiments with 48- and 72-hour–induced CEBPa-ER cells sorted into SSEA-1^low^ and SSEA-1^high^ fractions, using uninduced cells and TSCs as controls. Of a total of 59,548 (±11,990) regions with locally accessible chromatin (average of all samples tested), 9697 sites exhibited >2-fold changes after induction in at least one time point and cell fraction, denoted in the following as the “CEBPa regulome.” Most of the sites within this regulome showed stronger responses in CEBPa^high^ cells (fig. S7A), reflecting the observed dose dependence of gene expression and CEBPa binding. Unexpectedly, only about one-third of the sites (3,272) were found to be occupied by CEBPa. To study how CEBPa induces direct and indirect accessibility changes, we performed a motif analysis of the CEBPa regulome, showing enrichment of TEAD4 and ELF5 motifs at sites that opened but were not bound by CEBPa and of TEAD4 and TFAP2C motifs at opened and bound sites (Fig. 7A). In contrast, sites that closed were selectively enriched for POU5F1, SOX2, ESRRB, and NR5A2 motifs, regardless of CEBPa binding, while the KLF5 motif was enriched under all conditions (Fig. 7A).

Our findings show that CEBPa increases chromatin accessibility of putative TE-GREs by direct and indirect mechanisms. In addition, CEBPa mediates chromatin closing, likely by removing key pluripotency factors from ESC-GREs. Both the opening and closing of chromatin sites occur in a CEBPa dose-dependent manner.

### The CEBPa regulome consists of TE- and ESC-associated enhancers

Unsupervised hierarchical clustering of the 9697 sites comprising the CEBPa regulome resulted in four distinct clusters (Fig. 7B). (i) *TE-GREs*: 1136 sites opened after induction by CEBPa and also opened in TSCs, but not in ESCs. (ii) *ESC-GRE*s: 6204 sites became closed after CEBPa induction and were open in ESCs, but not in TSCs. (iii) Stem cell (*SC*)*-GRE*s: 1123 sites became closed but were open in both ESCs and TSCs. (iv) Dose-independent (*DIND*) sites: 1234 sites became opened with both low and high levels of CEBPa and were not accessible in either ESCs or TSCs. The chromatin accessibility changes of clusters i to iii are CEBPa dose dependent and largely independent of the time of induction.

We next integrated our ATAC-seq data with published ChIP-seq data (*35*, *82*–*85*) of TE and ESC-associated TFs’ bindings in TSCs and ESCs, respectively. This revealed a strong enrichment for the binding of TFAP2C, TEAD4, CDX2, and ELF5 at TE-GREs in TSCs and of OCT4, SOX2, NANOG, ESRRB, and KLF5 at ESC-GREs in ESCs. Notably, CEBPa preferentially bound to DIND sites and TE-GREs (Fig. 7B and fig. S7B). Our single-cell ATAC-seq data identified 390 DARs within the CEBPa regulome. Among these, DIND sites were enriched for ES2 to ES3 opening regions (fig. S7C), suggesting an early role during TELC formation. In addition, while most of TE-GREs opened during the ES3 to ET transition, most ESC-GREs closed at this step (fig. S7C).

### CEBPa-regulated TE-GREs are present in the gene body of TE-associated TFs

Because the ultimate drivers of cell fate specification are lineage-restricted TFs, we examined the genomic loci (300 kb) of the core TE-associated TFs (*Tead4*, *Tfap2c*, *Gata3*, *Eomes*, *Elf5*, *Id2*, and *Cdx2*) for the presence of TE-GREs regulated by CEBPa. This revealed a total of 16 TE-GREs (opening after CEBPa activation) distributed among the seven genes (Fig. 7C and fig. S7D). A similar analysis performed with pluripotency regulator loci revealed five ESC-GREs (closing sites) for *Sox2*, *Esrrb*, and *Pou5f1* and one TE-GRE for *Esrrb* (Fig. 7D and fig. S7E). The lineage affiliation of these sites was validated by their specific chromatin accessibility, H3K27ac decoration, and TF occupation in TSCs and ESCs, using published data (*35*, *82*–*86*). In addition, we detected a DIND site within the *Gata3* locus, not associated with either TE- or ESC-affiliated GREs (Fig. 7E).

In conclusion, our data show that CEBPa modulates chromatin accessibility at 9697 genomic sites, largely in a dose-dependent manner. These regions include lineage-specific GREs near core TE- and ESC-associated TFs, suggesting a role in their regulation. Moreover, CEBPa preferentially binds and opens DIND sites and TE-GREs, consistent with its reported pioneer factor activity in other cellular contexts (*7*, *87*).

### CEBPa-regulated TE-GREs are already either active or primed in early embryos

A central question emerging from our findings is whether putative GREs identified in response to CEBPa overexpression in ESCs are relevant to early embryonic development. To explore this, we analyzed the chromatin accessibility at the sites of the CEBPa regulome in two-, four- and eight-cell mouse embryos, i.e., before the TE-ICM bifurcation, using published ATAC-seq data (*36*). Among 3241 genomic regions that are closed at the two-cell stage but become accessible at the four- and eight-cell stage embryos, we identified 92 overlapping with the CEBPa regulome. Within this subset, DIND sites and TE-GREs were significantly enriched compared to SC- and ESC-GREs (fig. S8A), suggesting an early role for CEPBa-regulated elements.

**Fig. 8.**
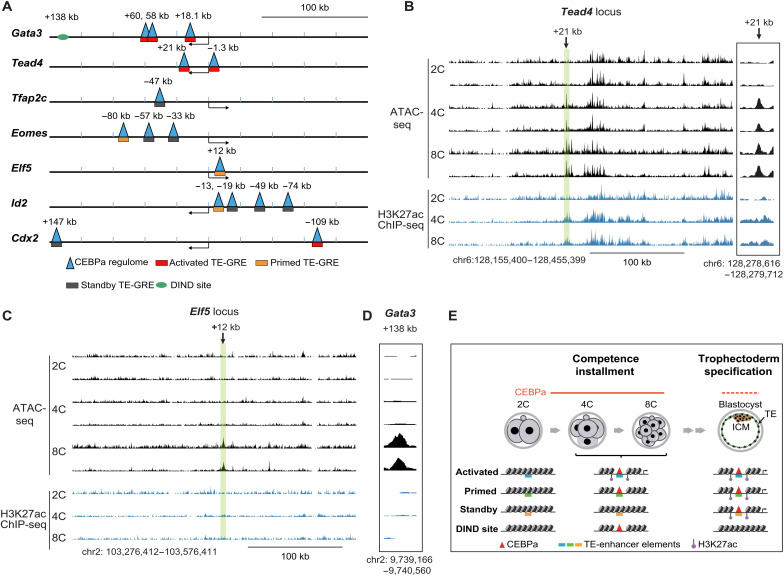
TE-GREs of key TE TFs are accessible in early embryos. (**A**) Summary of the distribution of GREs (putative enhancers) across the gene bodies of seven core TE-TFs spanning a 300-kb region, integrating ATAC-seq data obtained from CEBPa overexpression experiments and public data from embryos. The graph shows maps centered on the genes’ transcription start sites, with arrows pointing in the direction of transcription. The triangles denote sites opened after CEBPa overexpression in ESCs. The rectangles denote TE-GREs that are activated, primed, or standby, depending on their chromatin accessibility and H3K27ac decoration in four- and eight-cell embryos. DIND site is indicated by green. (**B** and **C**) Screenshots from the UCSC browser showing chromatin accessibility changes (*36*) and H3K27ac decoration (*123*) for the *Tead4* (B) and *Elf5* (C) loci in early embryos. Biological replicates are shown for the ATAC-seq samples. The activated and primed TE-GREs of *Tead4* (+21 kb) and *Elf5* (+12 kb), respectively, are highlighted with a green bar and correspond to the sites shown in Fig. 7C. 2C, two-cell; 4C, four-cell; 8C, eight-cell. (**D**) Screenshot from the UCSC browser showing chromatin accessibility changes (*36*) and H3K27ac decoration (*123*) for the DIND site at the *Gata3* locus shown in Fig. 7E. (**E**) Diagram depicting proposed changes during early embryo development of chromatin accessibility and enhancer mark decoration of sites within key TE-TF loci comprising the CEBPa regulome. The chromatin representations illustrate the four types of TE-associated regulatory regions identified in four- to eight-cell blastomeres: Activated enhancers are accessible and decorated with H3K27ac; primed enhancers are accessible but not decorated by the histone mark; standby enhancers are inaccessible and H3K27ac free, and DIND sites are accessible in four- and eight-cell embryos, but not in TSCs and ESCs.

We next examined the accessibility and enhancer activity of the 16 previously identified CEBPa-regulated TE-GREs at the four- or eight-cell stages. Of these, nine were accessible at one or both stages, while the remaining seven were closed and are hereafter referred to as “standby” TE-GREs (fig. S8B). Among the nine accessible TE-GREs, six were marked by H3K27ac and identified as activated enhancers (*88*), while three were classified as primed due to the absence of H3K27ac. The genomic distribution of these activated, primed, and standby TE-GREs, along with the DIND site, across the seven TE TF loci is shown in Fig. 8A. Notably, no additional activated or primed TE-GREs could be detected outside the CEBPa regulome within the aggregate 2.1 Mb comprising the seven TF loci, highlighting the specificity of CEBPa-associated enhancer activity during early lineage priming.

The +21 kb *Tead4* enhancer (Fig. 7C) exemplifies an activated TE-GRE that becomes both transposase-accessible and marked with H3K27ac in four- and eight-cell stage embryos. A view of the *Tead4* gene body shows the unique accessibility of this site within the 300-kb locus (Fig. 8B). In turn, the +12-kb *Elf5* TE-GRE exemplifies a primed enhancer, showing specific chromatin accessibility in eight-cell stage embryos but without H3K27ac enrichment (Fig. 8C). Its unique accessibility within the 300-kb locus matches the position of CEBPa binding and chromatin opening in induced ESCs (Fig. 7C). In addition, the +138-kb *Gata3* DIND site (Fig. 7E) was also accessible at the eight-cell stage (Fig. 8D).

Together, our results show that all enhancers selectively accessible in four- and eight-cell stage embryos at key TE-TF loci, whether activated or primed, are part of the CEBPa regulome. This suggests that CEBPa plays a central role in establishing a transcriptional landscape that endows early blastomeres with TE competence, before the TE-ICM bifurcation (Fig. 8E).

## DISCUSSION

Our data suggest that CEBPa is a so far unrecognized regulator with dual roles in the development of mouse embryos. Its expression first peaks in four-cell stage blastomeres and is later restricted to TE cells of the blastocyst. CEBPa ablation in zygotes delays morula formation in a subset of embryos and reduces the proportion of cells in the TE layer of blastocysts. Overexpression of CEBPa in ESCs induces the stepwise generation of TELCs, enabling the identification of CEBPa-regulated enhancers (the CEBPa regulome). A subset of the CEBPa regulome, located within the body of key TE-associated TF genes, becomes accessible as early as in four-cell embryos. Our findings suggest that the early role of CEBPa consists in the installment of TE competence before the TE-ICM lineage bifurcation. However, definitive proof will require the demonstration that CEBPa directly binds to and activates the relevant enhancers in four- to eight-cell embryos.

The detection of activated *Tead4*, *Gata3*, and *Cdx2* enhancers already in four- and eight-cell stage embryos raises the question as to whether they express the corresponding factors at subthreshold levels for TE commitment. Conversely, enhancers of *Eomes*, *Elf5*, *Tfap2c*, and *Id2*, which are only primed at the eight-cell stage, might correspond to genes activated during TE maturation (fig. S8C). The existence of standby enhancers, only activated late, suggests that an additional event is required for their activation in the TE. We speculate that this could involve the mysterious dosage independent sites, where low levels of CEBPa are already sufficient to open chromatin. This might trigger local chromatin restructuring that enables subsequent enhancer engagement at late activated enhancers, as we suggested for CEBPa-induced B cell reprogramming (*89*). Alternatively, late activation of TE-enhancers could depend on the delayed formation of enhancer-promoter loops, cofactor expression or broader architectural changes (*90*, *91*).

We also speculate that, after the acquisition of TE competence at the eight-cell stage, *Cebpa* is selectively down-regulated in inner cells destined to become ICM. Hippo signaling, which suppresses TEAD activity through nuclear YAP exclusion (*41*), may mediate this repression. In this framework, TEAD factors would sustain *Cebpa* expression in outer cells, while ICM formation would emerge as a default pathway. Furthermore, a transcriptional “memory” may be established at the four- to eight-cell stages that permits robust reexpression of *Cebpa* in outer cells forming the TE layer. Notably, the related factor CEBPb has been shown to induce trained immunity in hematopoietic stem cells (*92*), and Hippo signaling has been implicated in immune memory in *Drosophila* (*93*). In this context, the persistent localization of CEBPa on mitotic chromosomes (fig. S1E) may represent a bookmarking mechanism (*94*).

Our ESC model system captured two distinct populations of TELCs with different velocities of differentiation, depending on the level of CEBPa expression (fig. S8D). This points to a threshold mechanism, where cells surpassing a critical level of CEBPa are biased toward the TE fate, while cells with subthreshold levels retain plasticity. Extrapolating this idea to the embryo, CEBPa^high^ blastomeres in asymmetrically CEBPa-expressing four-cell embryos (fig. S1C) might be biased toward TE, in line with previous evidence for early fate bias (*25*, *27*, *29*, *95*, *96*). Consequently, cells with lower CEBPa levels would have a higher probability to engage in the ICM fate.

It has been shown that four- and eight-cell embryos express both TE- and ICM-associated TF genes, suggesting a bipotency state primed for cell fate resolution (*8*, *9*, *35*, *97*). This bipotency is thought to be governed by TFs, including NR5A2, TFAP2C, TEAD4, and KLF5, which activate genes of both TE and ICM lineages (*8*, *9*, *35*). Of note, CEBPa activates TE-restricted genes while silencing the ESC program, indicating that it predominantly acts in TE-lineage specification.

Unexpectedly, we found that SOX2, ESRRB, NR5A2, and KLF5 motifs are enriched not only in regions that close during differentiation but also open (Fig. 5D). This suggests a TF “switching” mechanism, in which the above factors translocate from ESC-GREs to TE-GREs, consistent with their known ability to bind both types of elements (*8*, *82*, *98*, *99*). Such TF relocations and repurposing have been described for T cell development, iPSC reprogramming, and B cell to macrophage transdifferentiation (*85*, *100*, *101*) and may cause fate oscillations between TE and ICM-like states. Supporting this, most accessible regions in the ET cluster contain binding motifs for both pluripotency and differentiation TFs (white areas in Fig. 5B). Perhaps relevant, oscillations between two cell fates have recently been reported for entero-endocrine progenitors during differentiation of the intestinal tract (*102*).

Our study raises the question as to whether CEBPA contributes to the priming of TE identity in human embryos. *CEBPA* mRNA expression has been reported to begin around E5 (fig. S1H). However, because ZGA in humans occurs at the eight-cell stage (*103*), it is plausible that key regulatory regions involved in TE priming begin to open during the late eight-cell to 16-cell stages, time points not covered in available datasets. Also, similar to the data presented here for mice, CEBPA overexpression in human ESCs activates the expression of TE-associated target genes (*104*). As such, the potential role of CEBPA in preparing human blastomeres for TE fate remains an intriguing possibility that warrants further investigation.

In summary, our findings expand the classical concept of developmental competence, traditionally focused on transcriptional priming (*105*), to include chromatin remodeling and subthreshold enhancer activation. Our results suggest that CEBPa unlocks a regulatory landscape that establishes TE competence and might contribute to the gradual restriction of totipotency during mouse embryo development.

## MATERIALS AND METHODS

### Mouse lines and embryos

For experiments involving preimplantation mouse embryos, B6CBAF1/Crl or C57Bl6xCBA females (purchased from Charles River Laboratories) were superovulated by injecting pregnant mare’s serum gonadotropin [100 μl of PMSG (50 IU/ml), Foligon] followed by human chorionic gonadotropin (100 μl, 50 IU/ml, Veterin Corion) after 48 hours. Females were then mated with B6CBAF1/Crl or C57Bl6xCBA males, respectively, and zygotes were harvested 20 hours after hCG injection from oviducts by puncturing ampullas in M2 medium (M7167, Sigma-Aldrich) containing hyaluronidase (300 μg/ml; H4272, Sigma-Aldrich) to remove the cumulus cells. The embryos were washed in M2 medium and subsequently in a few drops of Potassium Simplex Optimized Medium (KSOM) medium (MR106, Sigma-Aldrich) and cultured in KSOM microdrops under mineral oil (NO-100, Nidacon) in an incubator with 5% CO_2_ at 37°C. Embryos were handled with a mouth aspirator (A5177, Sigma-Aldrich) coupled to fire-polished glass Pasteur pipettes and collected at different stages of development from the in vitro cultures for protein immunostaining as detailed in the sections below.

To generate CEBPa KO embryos, microinjection was performed as previously described (*106*). Briefly, embryos were placed in a depression on a glass slide in M2 medium covered with mineral oil (Biocare Europe, 9305). Microinjection was performed using an Eppendorf Femtojet Microinjector with negative capacitance to facilitate membrane entry. The following RNA concentrations were used in microinjection mixes: *Cas9* mRNA (100 ng/μl), single guide RNAs (sgRNAs; 25 ng/μl), and *Gap43-RFP* RNA (50 ng/μl). After microinjection, the embryos were allowed to recover in M2 at 37°C and subsequently transferred into preequilibrated KSOM drops covered with mineral oil for long-term culture at 37°C and 5% CO_2_. Embryos were cultured to eight-cell stage and assessed for *Cebpa* expression by RT-qPCR to validate the knock out, or to the E4.5 blastocyst stage when wild-type and KO blastocysts were immunostained with CEBPa antibody and imaged to determine the percentage of embryos with a CEBPa ablation and whether the effect of the KO is heterogeneous or evenly distributed. Each experiment was performed at least in triplicate with a minimum of 19 embryos per group.

Mice were housed in standard cages under 12 hours of light-dark cycles and fed ad libitum with a standard chow diet. All experiments completed in Spain were approved by the Ethics Committee of the Barcelona Biomedical Research Park and performed according to Spanish and European legislation. For the experiments completed in the United Kingdom, this research adhered to the regulations of the Animals (Scientific Procedures) Act 1986 - Amendment Regulations 2012 and was reviewed by the University of Cambridge Animal Welfare and Ethical Review Body and approved by the Home Office (project license number PP3370287).

### Human embryos

Supernumerary embryos were obtained from patients undergoing standard intracytoplasmic sperm injection cycles. All embryos used for this research were donated with patients’ written informed consent, following cryopreservation. Blastocysts vitrified on day 5.5 were warmed using the Kitazato Warming Kit (VT602UF, Kitazato-Dibimed), as per the manufacturer’s instructions. Following warming, embryos were cultured to day 6.5 (*n* = 3) in 25-μl drops of continuous single IVF culture medium (CSCM, 90164, Irvine Scientific) under mineral oil (OVOIL, 10029, Vitrolife), at 37°C, 6% CO_2_, and 5% O_2_ (balance N2). Blastocyst quality was evaluated using the Gardner and Schoolcraft grading system, before fixation. Embryos were briefly exposed to prewarmed Acidic Tyrode’s Solution (T1788, Sigma-Aldrich) for removal of the zona pellucida. Blastocysts were handled using a 300-μm Flexipet pipette tip (K-FPIP-1300-10BS, COOK).

Approval for the use of human embryos was obtained from the Ethics Committee for Clinical Research of Clínica Eugin, Barcelona, Spain (CEIm EUGIN, project code EMBRYOTEST), and The Spanish National Commission for Human Reproduction (Comisión Nacional de Reproducción Humana Asistida, CNRHA, 0336S/2463/2018).

### Cell lines and culture conditions

ESCs and the CEBPa-ER cell lines were cultured on gelatinized plates in KnockOut DMEM (10829018, Gibco) containing 15% ESC fetal bovine serum (FBS; 16141079, Gibco), 1× penicillin-streptomycin (15140122, Gibco), 1× l-glutamine (25030081, Gibco), 1× nonessential amino acids (11140068, Gibco), 1 nM sodium pyruvate (11360070, Gibco), 0.1 mM 2-mercaptoethanol (31350010, Gibco), and fresh leukemia inhibitory factor (1000 U/ml) (ESG1106, Sigma-Aldrich). For CEBPa induction, cells were treated with 1 μM tamoxifen (H7904, Sigma-Aldrich), which shuttles the factor into the cell nucleus.

Mouse TSCs were established in J. Rossant’s laboratory (University of Toronto, Canada) and provided by M. Irimia (Centre for Genomic Regulation, Barcelona, Spain). TSCs were cultured in feeder-free conditions, where the basal medium was mixed with the conditioned medium in a 3:7 proportion. The basal medium consists of RPMI 1640 medium (12633012, Gibco) supplemented with 20% FBS (10270106, Gibco), 1× penicillin-streptomycin, 1× l-glutamine, 1× nonessential amino acids, 1 nM sodium pyruvate, and 0.1 mM 2-mercaptoethanol. Conditioned medium was obtained from mitotically inactivated mouse embryonic fibroblasts (house-made) cultured in the basal medium, collecting supernatants from days 3 to 9 of culture and cleared through 0.45-μm filters (SLHV033R, Millex). Upon TSC seeding, FGF4 (25 ng/ml; 235-F4, R&D System) and heparin (1 μg/ml; 3149, Sigma-Aldrich) were added to prevent differentiation.

All cell lines were incubated at 37°C and 5% CO_2_ in normoxia, and routinely passaged using trypsin when they reached 70% confluency. Mycoplasma tests were performed monthly to exclude contamination.

### Guide RNA design and testing

For targeted CEBPa KO in mouse embryos, target sequences for CRISPR-Cas9 were selected both upstream and downstream of the CEBPa coding sequence to ensure functional KO. CRISPR RNAs (crRNAs) were designed to be 17 to 21 nucleotides in length considering minimal off-target predictions and high on-target scores, as well as the immediate presence of protospacer adjacent motif sequences (NGG) and GC content. Various crRNA candidates were designed for each target region.

Each crRNA was cloned into a crRNA expression cassette (px330 plasmid) containing a fluorescent marker [either green fluorescent protein (GFP) or mCherry]. Mouse ESCs (E14) were transfected with each crRNA plasmid individually using the Amaxa Mouse ES Cell Nucleofector Kit (VPH-1001, Lonza). We used the A-024 program provided by the manufacturer after testing in the selected cell line. Cells were cultured again as previously described. After 8 to 24 hours, fluorescent cells were FACS sorted and cultured under normal conditions. After 48 hours, genomic DNA was extracted, the targeted regions were amplified through PCR, and the targeting efficiency of the individual crRNAs was assessed using an Alt-R Genome Editing Detection Kit (1075932, Integrated DNA Technologies) and agarose gel electrophoresis. The most efficient crRNAs were selected and verified by double transfection, FACS, and genotyping, following a similar procedure as described above.

Selected crRNAs:

CEBPa KO crRNA upstream: GGAGUCGGCCGACUUCUACG

CEBPa KO crRNA downstream: GCCAUGGGCAACUGCGCGTG

#### mRNA and gRNA preparation

For in vitro transcription, pRN3P-Gap43-RFP and pRN3P-Cas9 (gift from J. Na, Tsinghua University) plasmids were used. The constructs were linearized by restriction digestion downstream of the poly-A sequence and purified with QIAquick PCR purification kit (28104, QIAGEN). Subsequently, linearized plasmids were used for in vitro transcription using the mMessage mMachine T3 kit (AM1348, Thermo Fisher Scientific). mRNA was purified with lithium chloride precipitation, according to the manufacturer’s instructions.

sgRNAs targeting *Cebpa* were synthesized with a GeneArt gRNA kit (A29377, Thermo Fisher Scientific) by amplification of the DNA fragment containing T7 promoter, crRNA, and sgRNA sequence according to the manufacturer’s instructions. Subsequently, sgRNAs were in vitro transcribed and purified according to the manufacturer’s instructions.

### Generation of inducible CEBPa overexpressing cell lines

Briefly, lentiviruses were produced by transfecting human embryonic kidney–293 T cells with phage Ef1a-CEBPa-ERT2-IRES-dTomato (sequence available upon request) and pCMV-VSV-G, pCMVDR-8.91 plasmids using the calcium phosphate transfection method. Supernatant harvested 48 hours after transfection was filtered through 0.45-μm strainers and used for infection of ESCs, cultured for 3 days as described above, and subsequently single-cell FACS sorted on the basis of dTomato expression. After the expansion, two clones with high and homogeneous dTomato expressions were selected for further studies.

### Immunostaining

For embryos, samples were fixed in 4% paraformaldehyde (PFA) for 10 to 20 min at room temperature, followed by twice phosphate-buffered saline (PBS) washing for 5 min each before permeabilization with 0.5% Triton X-100/Tween 20 PBS (0.5% PBST). Zygote to morula stage embryos were permeabilized for 10 min, while mouse blastocysts were permeabilized for 15 min and human blastocysts for 20 min. Embryos were washed twice in 0.1% PBST for 5 min and then incubated in 0.1% PBST containing 3% bovine serum albumin (BSA) (A3294, Sigma-Aldrich) for 45 min at room temperature to block unspecific immunostaining. Primary antibodies were diluted in 0.1% PBST containing 1% BSA and added to the samples to incubate overnight at 4°C inside a moistened chamber. The next morning, embryos were sequentially washed in 0.1% PBST for 5, 15, 20, and 30 min at room temperature. A second blocking was performed in 0.1% PBST containing 3% BSA for 45 min at room temperature. For the secondary staining, embryos were placed in 0.1% PBST containing 1% BSA with the corresponding antibodies and 4′,6-diamidino-2-phenylindole (DAPI; 5 μg/ml; D1306, Thermo Fisher Scientific). Embryos were incubated in a secondary staining solution for 90 min at room temperature inside a moistened chamber in the dark. Three washes in 0.1% PBST were performed before mounting the embryos in 10 μl of PBS on 35-mm cover glass plates (P35G-1.0-14-C, MatTek) covered in light oil (M5310, Sigma-Aldrich). All the incubation steps were performed on shaking platforms.

For cultured cells, they were dissociated with trypsin and distributed in 12-well plates containing 0.01% poly-l-lysine–treated coverslips (72292-01, Electron Microscopy Sciences). The plates were centrifuged at 300 relative centrifugal force for 5 min to let the cells attach. The supernatant was removed and the cells were washed once with PBS before 4% PFA fixation of 20 min. Cells were then washed twice with PBS and permeabilized with 0.1% Triton X-100 PBS for 15 min at room temperature. Primary antibodies diluted in 0.1% PBST containing 1% BSA were added to the samples for overnight incubation. The next day, samples were washed twice with PBS and incubated with the corresponding secondary antibodies in the dark for 1 hour. Coverslips carrying the attached cells were then recovered with tweezers and mounted upside down onto a charged glass slide containing a 10-μl drop of mounting medium containing DAPI (F6057, Sigma-Aldrich). Coverslips were sealed with nail polish. Samples were imaged in a Leica SPE or SP5 inverted confocal microscopes and further processed in Fiji software.

### Flow cytometry

Cultured cells were dissociated with trypsin and resuspended in 100 μl of PBS containing mouse Fc block (1 μg/ml; 553141, BD Pharmingen) for 10 min. Conjugated SSEA-1 (50-8813-42, Cell Signaling Technology) or SCA-1 (25-5981-82, Invitrogen) antibodies were added to the blocking solution, and cells were further incubated on ice in the dark for 20 min. Cells were washed with 500 μl of PBS and centrifuged at 300 rcf for 5 min. The supernatant was discarded and the pellet was resuspended in 300 μl of PBS containing DAPI (5 μg/ml). Samples were processed in a FACS analyzer (Fortessa, BD) or sorter (Aria, BD). Data was analyzed by FlowJo software.

### Phagocytosis assay

A total of 200,000 cells were seeded per well in 12-well plates and cultured overnight in the presence of 1:1000 diluted carboxylated microspheres (17458-10, Fluoresbrite) added to the culture medium. For visualization, cells were processed for immunostaining as described above, with actin filaments stained with 1:100 diluted phalloidin (A12380, Thermo Fisher Scientific) and DNA stained with 1:500 diluted Quant-iT PicoGreen (P11495, Thermo Fisher Scientific). To quantify, cells were analyzed using an indo violet laser to detect the blue emission from the carboxylated microspheres.

### RNA extraction and RT-qPCR

For embryos, total RNA isolation was performed with Arcturus PicoPure RNA isolation kit (12204-01, Applied Biosystems) and reverse transcribed into cDNA using a High-Capacity RNA-to-cDNA Kit (4387406, Applied Biosystems) according to the manufacturer’s instructions. The Power SYBR Green RNA-to-CT 1-Step Kit (4389986, Applied Biosystems) was used to perform RT-qPCR, and experiment was performed with a StepOne Plus Real-time PCR machine (Applied Biosystems).

For cell lines, total RNA from the cell pellet was extracted using the RNeasy Mini Kit (74104, QIAGEN) and reverse transcribed as described above. RT-qPCR was performed in technical triplicate reactions using SYBR Green qPCR Master Mix (A25742, Applied Biosystems) and run in a Viia7 Real-Time PCR instrument (Applied Biosystems). Relative expression levels of target genes were calculated by the comparative cycle threshold method ΔΔCt using *Actb* or *Gapdh* as an endogenous reference gene for internal normalization.

### Cell proliferation assay

Cell proliferation was assessed using the CellTrace Violet Cell Proliferation Kit (C34557, Invitrogen) following the manufacturer’s instructions. Briefly, 1 million CEBPa-ER cells were resuspended in 1 ml of PBS, incubated with 5 μM CellTrace Violet dye at room temperature for 20 min and then washed with 5 volumes of culture medium before seeding. Cells were then treated with 4-OHT or MYC inhibitor 10058-F4 (HY-12702, MedChemExpress) for 48 hours before flow cytometry analysis.

### Bulk RNA-seq

Two clones were used for RNA-seq as biological replicates. Total RNA was extracted using the RNeasy Mini Kit (74104, QIAGEN), and libraries were prepared with a TruSeq Stranded mRNA Library Preparation Kit (20020595, Illumina) and assessed using Bioanalyzer DNA 1000 (5067-1504, Agilent). Libraries were sequenced on a HiSeq2500 instrument (Illumina), obtaining at least 40 million reads per sample.

### Bulk ATAC-seq

Two clones were used for ATAC-seq as biological replicates and the procedure was performed as described before (*70*). Briefly, 50,000 cells were harvested, washed with PBS, and resuspended in 50 μl of cold lysis buffer. After the centrifuge, the cell pellet was kept and proceeded for the Tn5 transposase TDE1-mediated transposition reaction (15027865, Illumina) at 37°C for 30 min, followed by DNA purification using a QIAGEN MinElute PCR Purification Kit (28004, QIAGEN). For library preparation, the transposed DNA fragments were amplified by PCR to add the sequencing barcodes and then purified using an AMpure reagent (A63880, Beckman). The concentration of libraries was measured by Qubit kit (Q32851, Thermo Fisher Scientific) and further diluted to 1.8 pM after denaturation. A pair-end sequencing aiming for 40 million reads per sample was run in the NextSeq instrument.

### Chromatin immunoprecipitation sequencing

For ChIP-seq, 10 million or 2 million CEBPa-induced cells were collected for the bulk sample and the SSEA-1 sorted fractions, respectively. The samples were crosslinked in 1% formaldehyde under rotation for 10 min, followed by glycine-mediated quenching. After centrifugation, the cell pellets were resuspended in SDS lysis buffer for 15 min and sonicated for 18 cycles of 30 s on and 30 s off using the Bioruptor Pico (Diagenode). The supernatant was incubated with protein A magnetic beads (10002D, Invitrogen) precoupled with CEBPa antibody (8718, Cell Signaling Technology) at 4°C overnight. The next day, DNA was collected after sequentially washing and elution, followed by proteinase K treatment and decrosslinking. Libraries were prepared using the DNA Library Prep for Illumina kit [E7370, New England Biolabs (NEB)] and amplified by Multiplex Oligos for Illumina kit (E6440, E6442, E6444, and E6446, NEB). Libraries were sequenced on the NextSeq2000 instrument (Illumina), obtaining a minimum of 40 million reads per sample.

### scRNA and scATAC-seq (multiomics)

A total of 1,000,000 cells of CEBPa-ER ESCs induced for 0, 3, 12, 48, and 72 hours, as well as TSCs, were trypsinized and resuspended in PBS containing 0.05% BSA. Libraries were prepared using the Chromium Single Cell Multiome ATAC + Gene Expression kit (1000283, 10x Genomics). Between 6400 and 14,800 cells per sample were sequenced using an Illumina NovaSeq6000 sequencer.

### Image processing

Image stacks were processed with the R package EBImage (*107*). Briefly, the DAPI channel was smoothed with a Gaussian filter of 9-pixel size and a sigma of 5. The DAPI channel was subsequently Otsu-thresholded to detect nuclei per image stack. Nuclear volumes corresponding to the same nucleus were stitched across stacks. Last, the average CDX2, CEBPA, and ELF5 intensity was calculated per nucleus.

### Computational analyses of bulk RNA-seq data

Reads were mapped using STAR (standard options) (*108*) and the Ensembl mouse genome annotation version mm10vM21. Gene expression was quantified using STAR (–quantMode GeneCounts). Sample scaling and statistical analysis were performed using the R package DESeq2 (R 3.3.2 and Bioconductor 3.0) (*109*). Statistical power of gene expression variation at any given time point was identified using the nbinomLRT test. Log_2_-vsd (variance stabilized DESeq2) counts were used for further analysis unless stated otherwise.

### Bulk ATAC-seq and ChIP-seq data processing

Raw reads were trimmed using TrimGalore (Cutadapt version 0.4.2_dev) (*110*). Reads were then aligned to the mm10 genome using bowtie2 (version 2.2.4) (*111*) with default parameters. Samtools (version 1.9) (*112*) were used to convert sam files to bam files that were then sorted and indexed using samtools view, sort, and index respectively. Nonprimary and supplementary alignments, alongside reads with a mapping quality score < 10, were filtered out using the samtools view -F 2304 -b -q 10 command. Read duplicates were detected and removed via the MarkDuplicates command of Picard-tools (https://broadinstitute.github.io/picard/, version 2.3.0). Deeptools bamCoverage (version 3.0.1) (*113*) was used to generate bigwig track files for visualization with the following options used:

1) ChIP-seq samples: --binSize 1 --normalizeUsing RPKM --effectiveGenomeSize 2150570000 --extendReads 147

2) ATAC-seq samples: --binSize 1 --normalizeUsing RPGC --effectiveGenomeSize 2150570000 --extendReads --outFileFormat bigwig

Peak calling was performed using MACS2 (version 2.1.1.20160309) (*114*) and default parameters. Motif analyses were carried out using tools of the MEME suite (*115*).

#### ChIP-seq downstream analysis

To identify differentially bound CEBPa sites between SSEA-1^low^ and SSEA-1^high^ cells, diffbind was used (*116*). A site was considered differentially bound if it had a false discovery rate (FDR) *P* value ≤ 0.05 and a |log_2_ fold change (log_2_FC)| ≥ 1. Sites with an average of <20 read counts across conditions were discarded.

#### ATAC-seq downstream analysis

To determine differentially accessible peaks, we compared 0-hpi ATAC-seq peak counts with all other 48- and 72- samples in pairs using diffbind. ATAC-seq peaks with an FDR-adjusted *P* value of ≤0.05 were deemed as differentially accessible. A final list of differentially accessible sites was then generated by merging all the pairwise differentially accessible sites using the bedtools merge command.

Normalized count values for the merged differentially accessible sites were obtained using diffbind. Differentially accessible sites were then plotted using the pheatmap R package and clustered via hierarchical clustering (clustering_method = “ward.D2,” clustering_distance_rows = “correlation”). Four final clusters of differentially accessible sites were pinpointed.

To determine chromatin-accessible peaks from the two- to eight-cell ATAC-seq data (*36*), we used a different peak calling strategy. Bam files were converted to Bed files using the bamToBed bedtools suite command. MACS2 was then used on the BED files with the following parameters: -g mm -f BED --nomodel --shift -75 --extsize 150.

### Computational analyses of scRNA- and scATAC-seq data (multiomics)

We obtained the transcript count and peak accessibility matrices using Cell Ranger Arc (v.1.0.0). Preprocessing was performed with Seurat (v.4.0.3) (*117*) and Signac (v.1.2.1) (*118*) R packages (GitHub). As a quality control, we filtered out the cells that fulfilled any of the following conditions: <800 RNA reads; >40,000 RNA reads; >10% mitochondrial RNA reads; <120 detected genes; >5500 detected genes; > 100,000 ATAC reads; <500 ATAC reads; TSS enrichment score < 2; and nucleosomal signal score > 2.5. To remove unwanted variability, we filtered out a set of housekeeping, cell cycle, and ribosomal genes from the RNA count matrix (a list of genes excluded is provided in table S1). Transcript and peak counts were normalized using log and term frequency-inverse document frequency (TD-IDF) normalization, respectively.

The preprocessing of the samples consisted of the following steps: We identified the HVGs using the FindVariableFeatures, followed by data scaling, and PCA using the default parameters of ScaleData and RunPCA functions of Seurat. The ESC and TSC samples were integrated using the Harmony software (v.0.1.0) (*77*) using the first 30 principal components of the RNA processed data. We then produced a shared 2D UMAP embedding with the main eight harmony dimensions.

### Clustering and cell annotation

For cell annotation, we first clustered the integrated dataset with the FindClusters function of Surat at a resolution of 0.1, which separated the cells into ESC-like cells and TSC-like cells, as indicated by marker genes. Then, each of these ESC-like and TSC-like clusters were reintegrated with Harmony and reclustered with a resolution of 0.35 and 0.1, respectively.

### Integration of E14 ESC cell sample

To identify the cluster that represents the initial cell state of the differentiating ESC cells, we used a label transfer method using our integrated dataset as reference and published E14 sample as a query dataset. The counts matrix of the E14 sample was obtained from the Gene Expression Omnibus (GEO) database (*78*) and preprocessed using the standard Seurat pipeline. We then used the FindTransferAnchors and MapQuery functions from Seurat. We also performed an RNA velocity analysis on the 12-hpi ESC sample using the default parameters of the generalized dynamical model of the scVelo python toolkit (*119*).

### Integration with physiological stages and CeLaVi

For further characterization of our clusters, we used published scRNA-seq data on mouse preimplantation embryos (*11*). More specifically, we downloaded the loom file from the GEO database (GSE145609) and preprocessed with the standard Seurat pipeline. Then, from the public dataset, we used cells annotated as “16cell,” “4cell,” “8cell,” “TE,” “late2cell,” “mid2cell,” “E3.0-3.5,” “E2.5-E2.75,” “E4.5,” and “PrE” to integrate with our 72-hpi sample. Before integration, we reannotated the cells from clusters “mid2cell” and “late2cell” as “2-cell”; “16cell” and “E2.5-E2.75” as “Morula”; “E4.5” as “Epiblast”; and “E3.0-3.5” as “ICM.” As the integration is sensitive to the relative number of cells in each cluster, we randomly selected 50 cells from our clusters so that the number of cells was comparable with the physiological clusters. We integrated the datasets using only common genes expressed in both datasets, using the FindIntegrationAnchors function from Seurat (with the parameters dims = 1:20 and k.filter = 80) and the IntegrateData function (parameters dims = 1:30 and weight = 50). We produced a common 3D embedding using the phate function (with knn = 30 and ndim = 30) of the PHATE R package (v.1.0.7) (*120*) and exported the 3D coordinates to visualize it with the CeLaVi visualization tool (*79*). For a quantitative estimation of the similarity of gene expression between our clusters and a given cluster in the public dataset, we resampled 10 times the same amount of cells and repeated the integration between datasets, computing in each iteration for each physiological cluster the *N*-nearest neighbors on the first 15 PCAs of the integrated dataset, where *N* was two times the number of cells in each cluster. Then, a similarity distance between a given cluster X from our dataset and a physiological cluster Y was calculated by dividing the number of cells of cluster X that is found in the nearest neighbors of cluster Y.

### Differential gene expression

We used the FindAllMarkers Seurat function to get the DEGs of each cluster against the rest of the cells, with the condition that the gene needed to be expressed in at least 10% of the cells of the interrogated cluster (min.pct = 0.1). We then randomly sampled 150 cells and used the top 10 DEG of each cluster to produce a heatmap with the scaled expression, using the package ComplexHeatmap (v.2.10.0) (*121*).

### Peak calling and analysis

For a more in-depth analysis of the single cell ATAC-seq data, we used the 72-hpi sample. First, we recalled the peaks with the CallPeaks function of Signac, which uses the algorithm macs3 (v3.0.0a6). Then, we quantified the counts in each peak with FeatureMatrix (Signac) and counts were tf-idf normalized.

### Motif analysis

To perform motif enrichment analysis, we first added the motif data from the JASPAR2020 database with the AddMotifs (Signac) function and the BSgenome.Mmusculus.UCSC.mm10 mouse genome from Bioconductor. We used the FindAllMarkers (Seurat R) function (with the options test.used = “LR” and only.pos = T) to get the DARs of each cluster against the rest of the cells. For all the DAR with an adjusted *P* value of <0.05, we tested for overrepresented motifs with the FindMotifs (Signac) function and generated a heatmap with the DoHeatmap (Seurat R) function and the per-cluster pseudo-bulk ATAC-seq data computed with the AverageExpression() function. For a more detailed analysis, we used the FindMarkers function (test.used = “LR”) to obtain the DAR between pairs of clusters following our hypothesized cluster transition and tested for overrepresented motifs in regions that opened and closed during this transition, identified by the positive and negative value of the log_2_FC value respectively. Last, we computed the per-cell motif activity with chromVAR (*81*), using the RunChromVAR (Signac) function.

## Supplementary Materials

### The PDF file includes:

Figs. S1 to S8 Legend for table S1 References
