# Supplementary material for: Role of CEBPa in trophectoderm competence installment
Source: Sci Adv. 2025 Nov 28;11(48):eady1693. doi: 10.1126/sciadv.ady1693 (PMC12662221; doi:10.1126/sciadv.ady1693)
Supplement: Supplementary file 1 — Figs. S1 to S8 Legend for table S1 References [file sciadv.ady1693_sm.pdf]

Science Advances
AAAS

Supplementary Materials for

Role of CEBPa in trophectoderm competence installment

Xiao Wei *et al.*

Corresponding author: Thomas Graf, thomas.graf@crg.eu; Magdalena Zernicka-Goetz, magdaz@caltech.edu

Sci. Adv. **11**, eady1693 (2025)
DOI: 10.1126/sciadv.ady1693

The PDF file includes:

Figs. S1 to S8
Legend for table S1
References

Other Supplementary Material for this manuscript includes the following:

Table S1

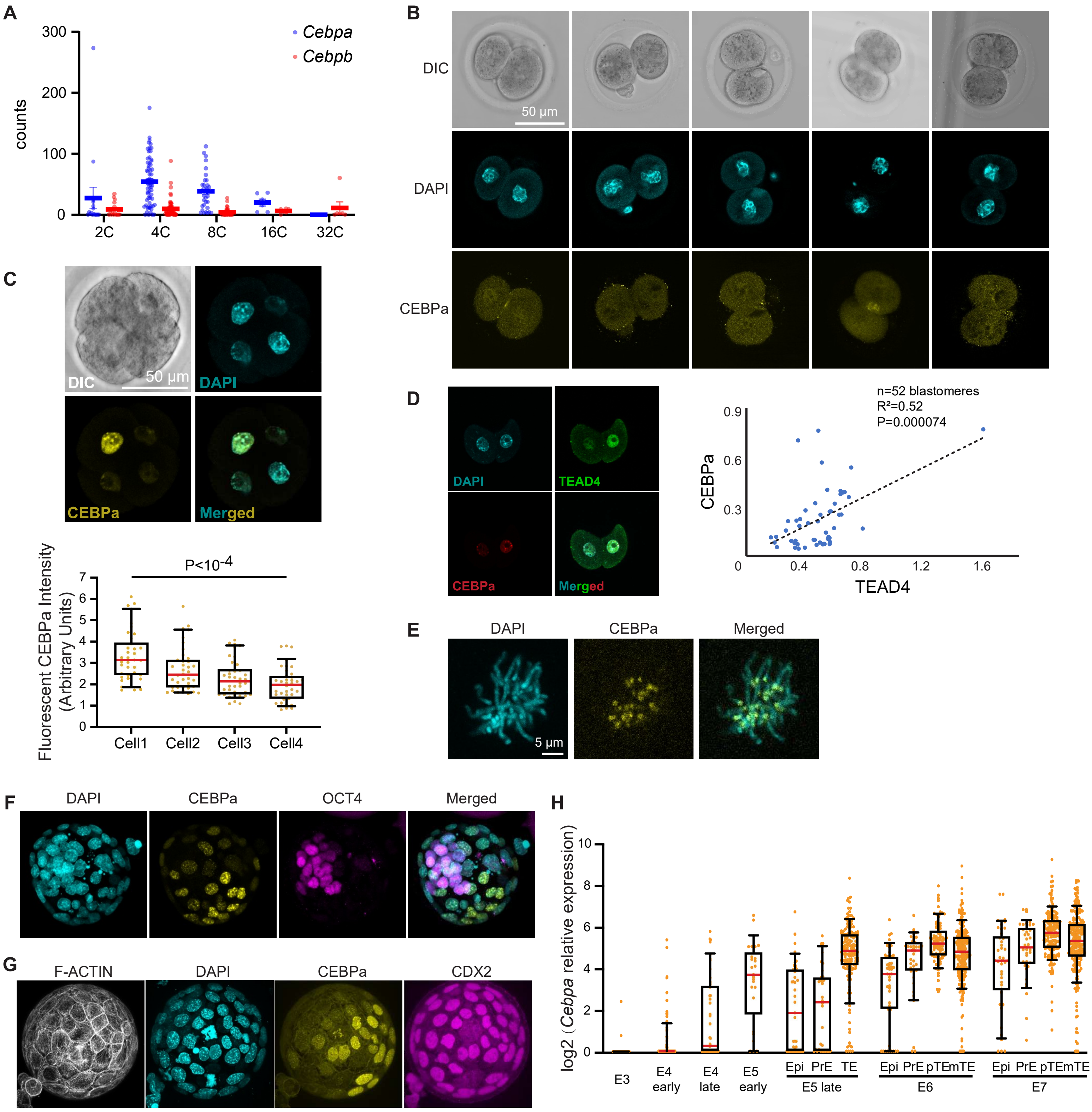
A
B
C
D
E
F
G
H
Cebpa
Cebpb
counts
2C
4C
8C
16C
32C
DIC
DAPI
CEBPa
50 µm
Merged
TEAD4
n=52 blastomeres
R²=0.52
P=0.000074
Fluorescent CEBPa Intensity (Arbitrary Units)
P<10-4
Cell1
Cell2
Cell3
Cell4
5 µm
OCT4
F-ACTIN
CDX2
log2 (Cebpa relative expression)
E3
E4 early
E4 late
E5 early
Epi PrE TE
E5 late
Epi PrE pTEmTE
E6
E7

Fig. S1. CEBPa becomes heterogeneously expressed at the 2- to 4-cell transition and in the trophectoderm lineage

(**A**) Expression of *Cebpa* and *Cebpb* in mouse embryos analyzed from public single-cell RNA-seq data (*27*).

(**B**) Immunostaining for CEBPa in 2-cell mouse embryos. Upper, DIC; middle, DAPI; lower, CEBPa.

(**C**) Left: representative 3D projection of CEBPa expression at the 4-cell stage measured by IF; Right: quantification of nuclear CEBPa intensity in 4-cell embryos (n=17) normalized by the cytoplasmic background for each blastomere.

(**D**) Left: representative immunostaining for CEBPa and TEAD4 in disaggregated blastomeres from 4-cell stage embryos; Right: quantification of nuclear CEBPa and TEAD4 intensities in blastomeres of 4-cell stage embryos normalized by the cytoplasmic background for each blastomere. One outlier was excluded.

(**E**) Immunostaining for CEBPa in the blastomere from a 4-cell embryo that entered mitosis (prophase).

(**F**) Immunostaining for CEBPa and OCT4 in a late blastocyst.

(**G**) Immunostaining for CEBPa and CDX2 in a late blastocyst. Note that the expression of CEBPa is restricted to a subset of TE cells.

(**H**) Expression of *CEBPA* (single-cell RNA-seq) in human preimplantation embryos (*63*). Boxplots and whiskers depict 10-90 percentiles with the red line indicating median values.

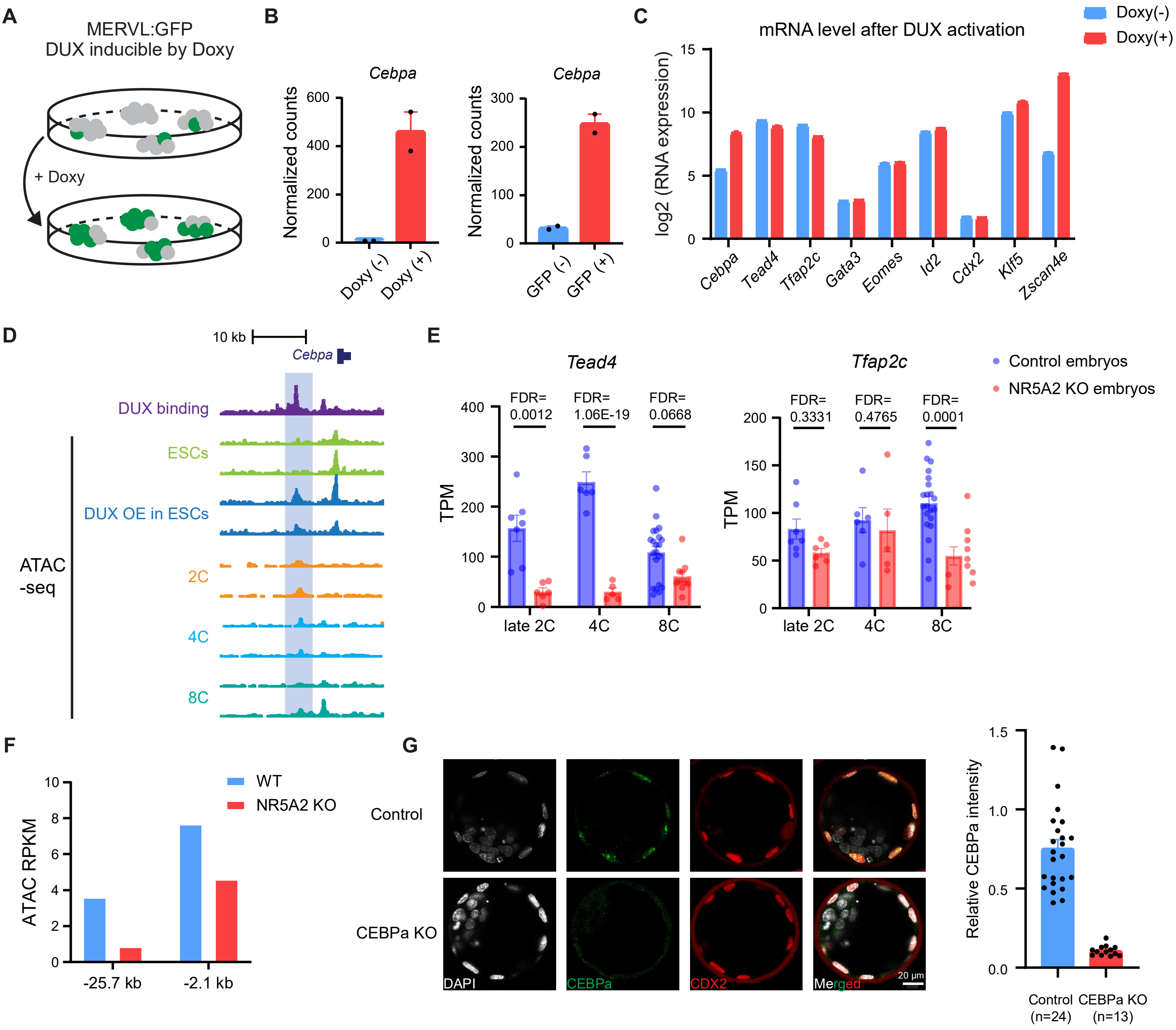
A
MERVL:GFP
DUX inducible by Doxy
+ Doxy
B
Cebpa
Normalized counts
Doxy (-)
Doxy (+)
GFP (-)
GFP (+)
C
mRNA level after DUX activation
log2 (RNA expression)
Doxy(-)
Doxy(+)
Cebpa
Tead4
Tfap2c
Gata3
Eomes
Id2
Cdx2
Klf5
Zscan4e
D
10 kb
DUX binding
ESCs
DUX OE in ESCs
ATAC
-seq
2C
4C
8C
E
Tead4
Tfap2c
Control embryos
NR5A2 KO embryos
FDR=
0.0012
FDR=
1.06E-19
FDR=
0.0668
FDR=
0.3331
FDR=
0.4765
FDR=
0.0001
TPM
late 2C
4C
8C
F
ATAC RPKM
WT
NR5A2 KO
-25.7 kb
-2.1 kb
G
Control
CEBPa KO
DAPI
CEBPa
CDX2
Merged
20 μm
Relative CEBPa intensity
Control
(n=24)
CEBPa KO
(n=13)

Fig. S2. NR5A2 regulates *Cebpa* expression

(**A**) Schematics of a reporter system where doxycycline (Doxy) mediated activation of DUX in mouse embryonic stem cells (mESCs) induces expression of a MERVL-GFP reporter and a 2-cell-like state (*69*).

(**B**) Changes of *Cebpa* mRNA levels after DUX activation of the MERVL-GFP reporter line (left) or in GFP-sorted (right) cells (*69*). Data are represented as mean ± SEM.

(**C**) Comparison of TE-associated TF gene expression between control (Doxy-) and DUX-induced (Doxy+) mESCs (*69*). Data are represented as mean ± SD

(**D**) Screenshot from the UCSC browser showing DUX binding at -7.1 kb of *Cebpa* in 24h DUX-induced mouse ESCs, highlighted in light blue. The same region shows increased chromatin accessibility not seen in ESCs (*69*). The following 6 lanes show ATAC-seq signals of 2- to 8-cell embryos (*36*), revealing accessible chromatin of the -7.1 kb region in late 2-cell and 4-cell embryos.

(**E**) Effect of zygotic NR5A2 knockout (KO) on *Tead4* and *Tfap2c* expression at late 2-cell, 4-cell and 8-cell stages (*66*).

(**F**) Effect of NR5A2 KO on the accessibility of two potential NR5A2-regulated enhancers upstream of *Cebpa* in 8-cell stage embryos as analyzed from public ATAC-seq (*66*).

(**G**) Efficiency of CEBPa KO by CRISPR/Cas9. Shown are representative IF images of blastocysts stained with CEBPa (green) and CDX2 (red), and quantification of CEBPa intensity in CEBPa knockouts compared to WT embryos (right).

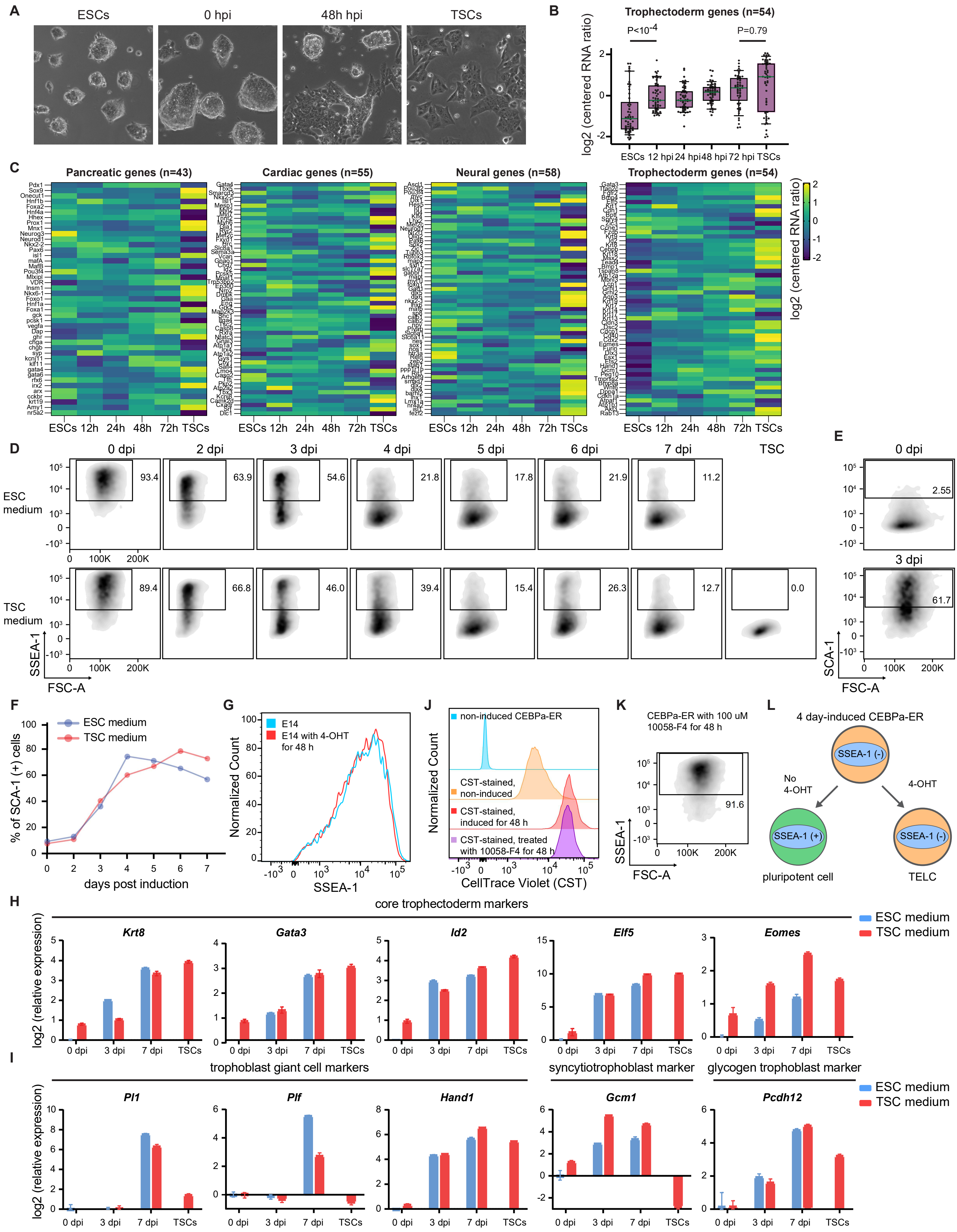
A
ESCs
0 hpi
48h hpi
TSCs
B
Trophectoderm genes (n=54)
P<10-4
P=0.79
log2 (centered RNA ratio)
ESCs 12 hpi 24 hpi 48 hpi 72 hpi TSCs
C
Pancreatic genes (n=43)
Cardiac genes (n=55)
Neural genes (n=58)
Trophectoderm genes (n=54)
ESCs 12h 24h 48h 72h TSCs
log2 (centered RNA ratio)
D
0 dpi
2 dpi
3 dpi
4 dpi
5 dpi
6 dpi
7 dpi
TSC
ESC medium
93.4
63.9
54.6
21.8
17.8
21.9
11.2
TSC medium
89.4
66.8
46.0
39.4
15.4
26.3
12.7
0.0
SSEA-1
FSC-A
E
0 dpi
2.55
3 dpi
61.7
SCA-1
FSC-A
F
ESC medium
TSC medium
% of SCA-1 (+) cells
days post induction
G
E14
E14 with 4-OHT for 48 h
Normalized Count
SSEA-1
J
non-induced CEBPa-ER
CST-stained, non-induced
CST-stained, induced for 48 h
CST-stained, treated with 10058-F4 for 48 h
Normalized Count
CellTrace Violet (CST)
K
CEBPa-ER with 100 uM 10058-F4 for 48 h
91.6
SSEA-1
FSC-A
L
4 day-induced CEBPa-ER
SSEA-1 (-)
No 4-OHT
4-OHT
SSEA-1 (+)
SSEA-1 (-)
pluripotent cell
TELC
H
core trophectoderm markers
ESC medium
TSC medium
Krt8
Gata3
Id2
Elf5
Eomes
log2 (relative expression)
0 dpi 3 dpi 7 dpi TSCs
I
trophoblast giant cell markers
syncytiotrophoblast marker
glycogen trophoblast marker
ESC medium
TSC medium
Pl1
Plf
Hand1
Gcm1
Pcdh12
log2 (relative expression)
0 dpi 3 dpi 7 dpi TSCs

Fig. S3. Ectopic expression of CEBPa in ESCs induces the formation of trophectoderm lineage cells

(**A**) Phase contrast images showing morphological changes in 48h CEBPa-induced ESCs, compared to E14 ESCs and TSCs.

(**B**) Expression changes of trophectoderm signature genes (n=54) after CEBPa induction in ESCs. TSCs were used as control. Dots represent average expression values for each gene among n=2 biological replicates. Boxplots and whiskers depict 10-90 percentiles with green lines indicating median values. Statistical significance was determined using two-way ANOVA and Tukey's multiple comparison tests.

(**C**) Heatmaps showing average expression of pancreatic (n=43), cardiac (n=55), neural (n=58), and trophectoderm (n=54) signature genes after CEBPa induction in ESCs, average from duplicates. TSCs were used as control.

(**D**) FACS plots of CEBPa-induced ESCs grown in ESC and TSC media analyzed for SSEA-1 expression and forward scatter (FSC-A). TSCs and uninduced ESCs were used as controls.

(**E**) Expression of the TE-specific cell surface marker SCA-1 in non-induced and 3 day-induced CEBPa-ER cells analyzed by FACS.

(**F**) Kinetics of SCA-1 positive cells in induced CEBPa-ER cultured in ESC and TSC media.

(**G**) Expression of SSEA-1 in E14 and E14 treated with 4-OHT for 48 hours.

(**H**) Core TE marker gene expression (RT-qPCR) after CEBPa induction in ESC and TSC media, with TSCs shown as a control. Ct values are relative to *Actb* and normalized to ESCs cultured in ESC medium. Data show mean ± SEM.

(**I**) Same analysis as (H) for marker genes of syncytiotrophoblasts, glycogen trophoblast cells, and trophoblast giant cells. Data are represented as mean ± SEM.

(**J**) Cell proliferation measured by the dilution of CellTrace Violet dye in non-induced, 4-OHT-induced, or MYC inhibitor 10058-F4-treated CEBPa-ER for 48 hours.

(**K**) Expression of SSEA-1 in CEBPa-ER treated with 10058-F4 for 48 hours.

(**L**) Diagram depicting the plasticity of early TELCs. SSEA-1 negative cells sorted from 4 dpi CEBPa-ER can either regain pluripotency upon withdrawal of the inducer or continue differentiating into mature TELCs with sustained CEBPa expression.

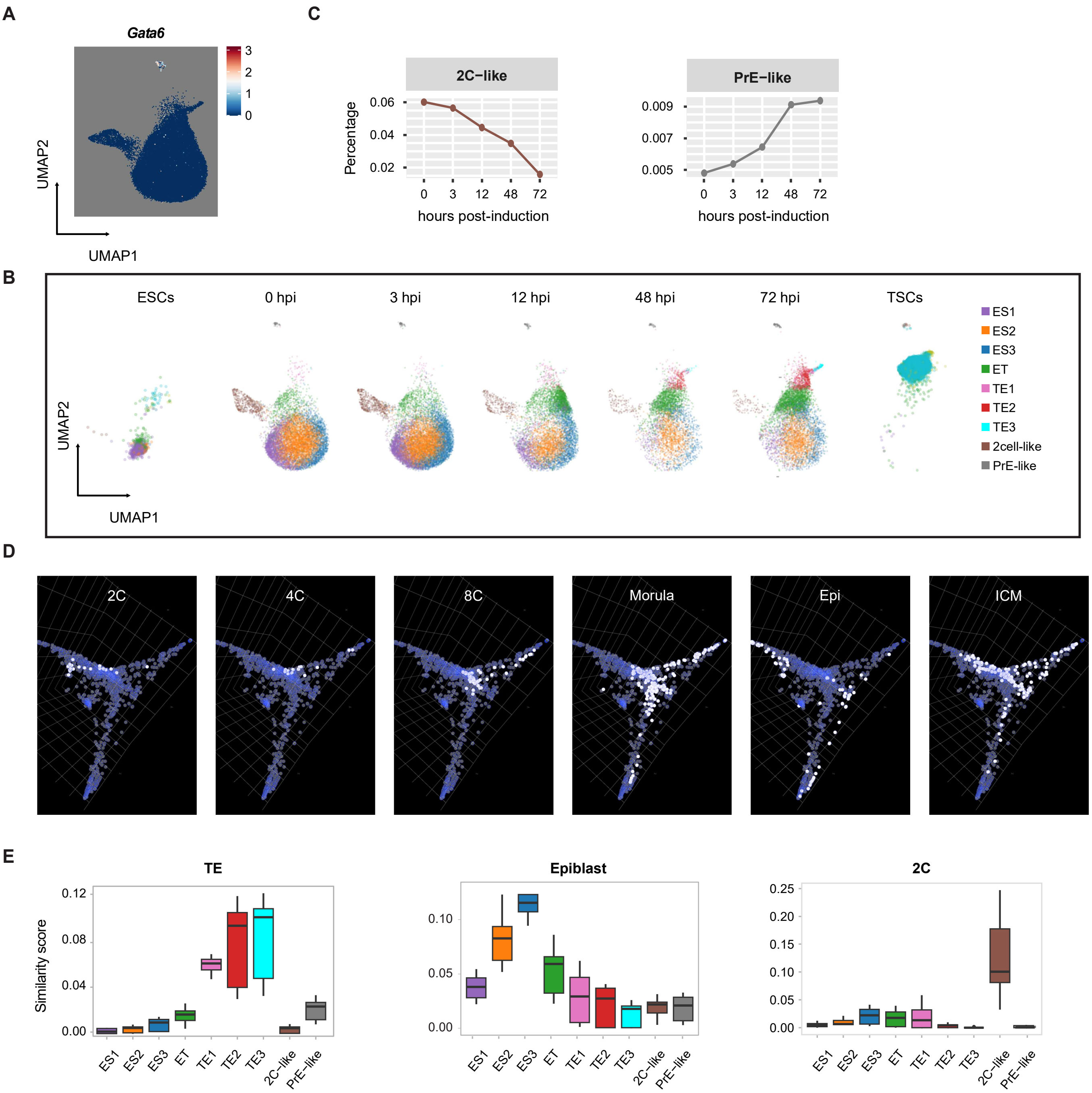
A
Gata6
UMAP2
UMAP1
C
2C−like
PrE−like
Percentage
hours post-induction
B
ESCs
0 hpi
3 hpi
12 hpi
48 hpi
72 hpi
TSCs
ES1
ES2
ES3
ET
TE1
TE2
TE3
2cell-like
PrE-like
D
2C
4C
8C
Morula
Epi
ICM
E
TE
Epiblast
2C
Similarity score
2C−like
PrE−like

Fig. S4. Single-cell analyses reveal progressive ESC to TE-lineage transitions and the stepwise activation of TE-associated genes

(**A**) UMAP with expression of the primitive endoderm marker *Gata6* (light dots in a small cluster on top of the figure) in the integrated samples shown in Fig. 4C.

(**B**) UMAPs showing the cell distribution at different time points, with ESCs and TSC as controls.

(**C**) Stacked bar plot showing the percentages of 2-cell-like and primitive endoderm-like clusters at each time point.

(**D**) Distribution of different embryo development stages in a 3D cell landscape visualized by CeLaVi.

(**E**) Comparison of transcriptional similarities between integrated embryo development stages (TE, epiblast, and 2-cell stage) and CEBPa-induced cells.

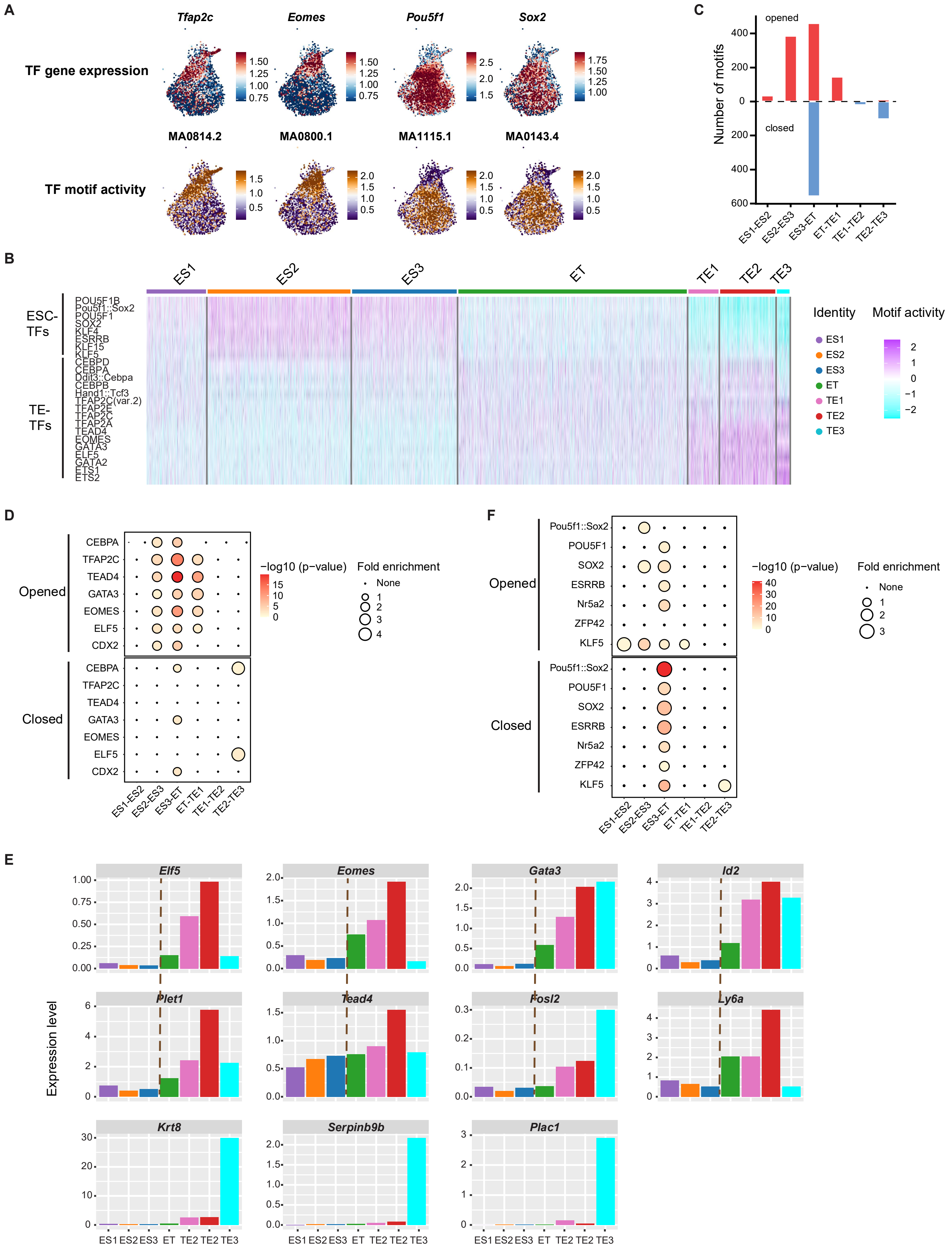

A
Tfap2c
Eomes
Pou5f1
Sox2
TF gene expression
MA0814.2
MA0800.1
MA1115.1
MA0143.4
TF motif activity
C
opened
closed
Number of motifs
ES1-ES2
ES2-ES3
ES3-ET
ET-TE1
TE1-TE2
TE2-TE3
B
ES1
ES2
ES3
ET
TE1
TE2
TE3
ESC-TFs
TE-TFs
POU5F1B
Pou5f1::Sox2
POU5F1
SOX2
KLF4
ESRRB
KLF15
KLF5
CEBPD
CEBPA
Ddit3::Cebpa
CEBPB
Hand1::Tcf3
TFAP2C(var.2)
TFAP2E
TFAP2C
TFAP2A
TEAD4
EOMES
GATA3
ELF5
GATA2
ETS1
ETS2
Identity
Motif activity
D
Opened
Closed
CEBPA
TFAP2C
TEAD4
GATA3
EOMES
ELF5
CDX2
−log10 (p−value)
Fold enrichment
None
F
Pou5f1::Sox2
POU5F1
SOX2
ESRRB
Nr5a2
ZFP42
KLF5
E
Elf5
Eomes
Gata3
Id2
Plet1
Tead4
Fosl2
Ly6a
Krt8
Serpinb9b
Plac1
Expression level
ES1 ES2 ES3 ET TE2 TE2 TE3

Fig. S5. CEBPa induces the priming and sequential activation of TE-associated enhancers

(**A**) Comparison of the gene expression of ESC and TE marker genes (upper) and their motif activity (lower) in the 72 hpi samples, quantified using the scRNA-seq and scATAC-seq data of the multi-omics respectively.

(**B**) Motif activities of selected ESC- and TE-associated TFs in the single-cell clusters observed after CEBPa induction.

(**C**) Number of motifs overrepresented in the DARs between cluster pairs in the ES1 to TE3 cluster transitions. Motifs that become accessible/ inaccessible are shown in red/ blue, respectively.

(**D**) Enrichment and its p-values for specific TE-associated motifs that open (top panels) or close (bottom panels).

(**E**) Expression kinetics of TE-specific genes in differentiating single-cell clusters. The stippled lines indicate the transition from clusters not expressing to clusters expressing the relevant genes.

(**F**) Enrichment and its p-values for specific ESC-associated motifs that open (top panels) or close (bottom panels).

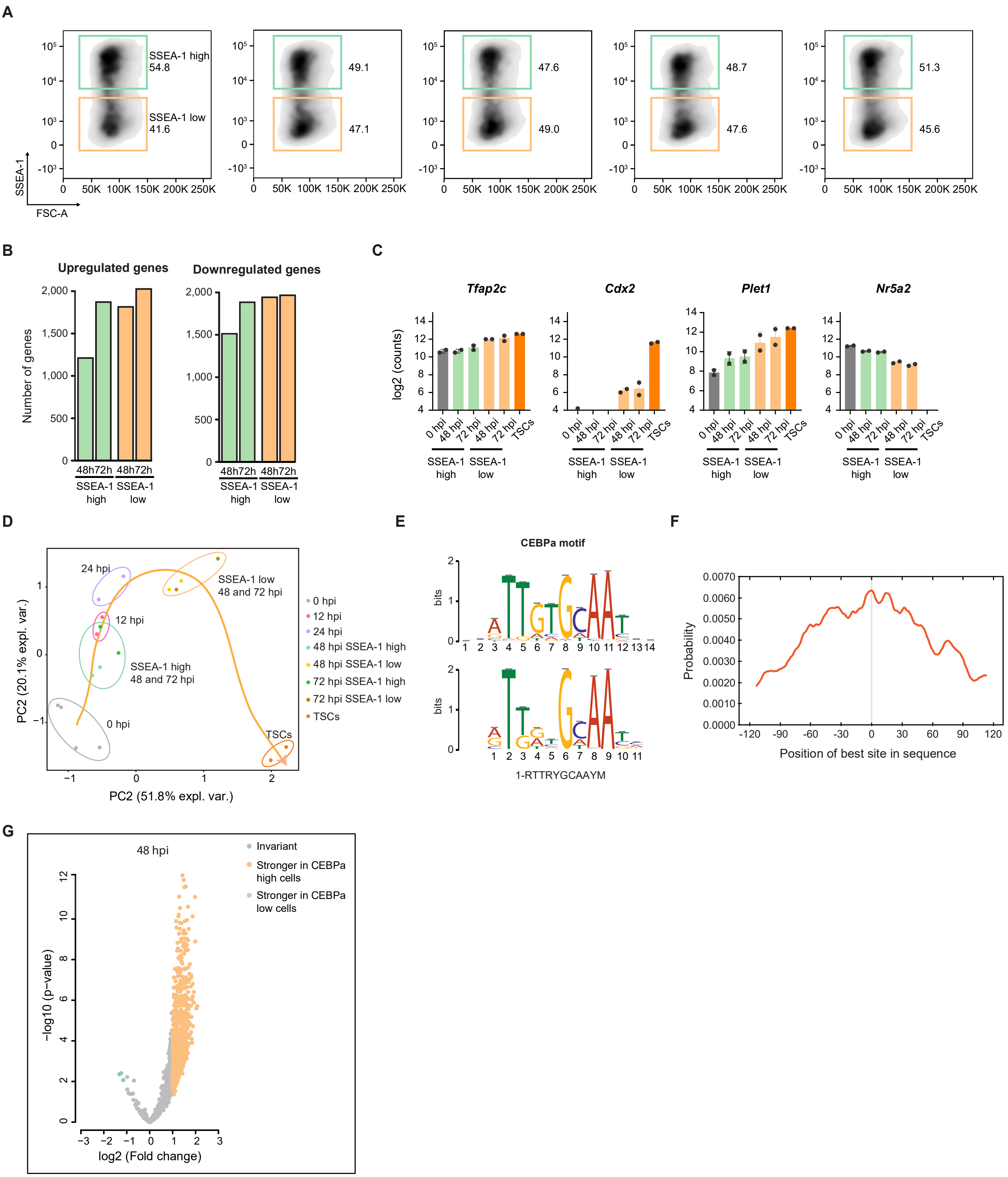
A
SSEA-1 high
54.8
SSEA-1 low
41.6
49.1
47.1
47.6
49.0
48.7
47.6
51.3
45.6
SSEA-1
FSC-A
0 50K 100K 150K 200K 250K
B
Upregulated genes
Downregulated genes
Number of genes
48h72h 48h72h
SSEA-1 high SSEA-1 low
C
Tfap2c
Cdx2
Plet1
Nr5a2
log2 (counts)
0 hpi 48 hpi 72 hpi 48 hpi 72 hpi TSCs
SSEA-1 high SSEA-1 low
D
24 hpi
12 hpi
SSEA-1 low 48 and 72 hpi
SSEA-1 high 48 and 72 hpi
0 hpi
TSCs
PC2 (20.1% expl. var.)
PC2 (51.8% expl. var.)
0 hpi
12 hpi
24 hpi
48 hpi SSEA-1 high
48 hpi SSEA-1 low
72 hpi SSEA-1 high
72 hpi SSEA-1 low
TSCs
E
CEBPa motif
bits
1-RTTRYGCAAYM
F
Probability
Position of best site in sequence
G
48 hpi
Invariant
Stronger in CEBPa high cells
Stronger in CEBPa low cells
−log10 (p−value)
log2 (Fold change)

Fig. S6. High and low levels of CEBPa drive the formation of two distinct TELC fractions

(**A**) FACS plots showing the formation of SSEA-1^{low} and SSEA-1^{high} fractions at 3 dpi from 5 different batches of ESCs induced with CEBPa in ESC medium. Percentages of cells in the gates shown are indicated.

(**B**) Numbers of differentially expressed genes identified in SSEA-1^{high} and SSEA-1^{low} cells derived at 48 and 72 hpi compared to uninduced cells.

(**C**) Average expression of lineage-restricted genes in sorted SSEA-1^{high} and SSEA-1^{low} cell fractions at 48 and 72 hpi, as determined by RNA-seq. Uninduced ESCs (0 hpi) and TSCs shown as controls. Dots represent expression in 2 biological replicates.

(**D**) Principal component analysis (PCA) of gene expression dynamics after CEBPa induction, showing biological duplicates (circled) and an approximate average trajectory. TSCs are used as a control and endpoint.

(**E**) Comparison of CEBPa motif (MA0102.4) deposited in the JASPAR database (upper) with the top motif identified from CEBPa ChIP-seq peaks in 24 hpi cells (lower).

(**F**) Site distribution of the enriched CEBPa motif in the peaks called from ChIP-seq data.

(**G**) Comparison of CEBPa binding between CEBPa^{low} and CEBPa^{high} cells at 48 hpi. Fold change = binding signal (CEBPa^{high} cells/ CEBPa^{low} cells). P-values were calculated by DiffBind (*122*).

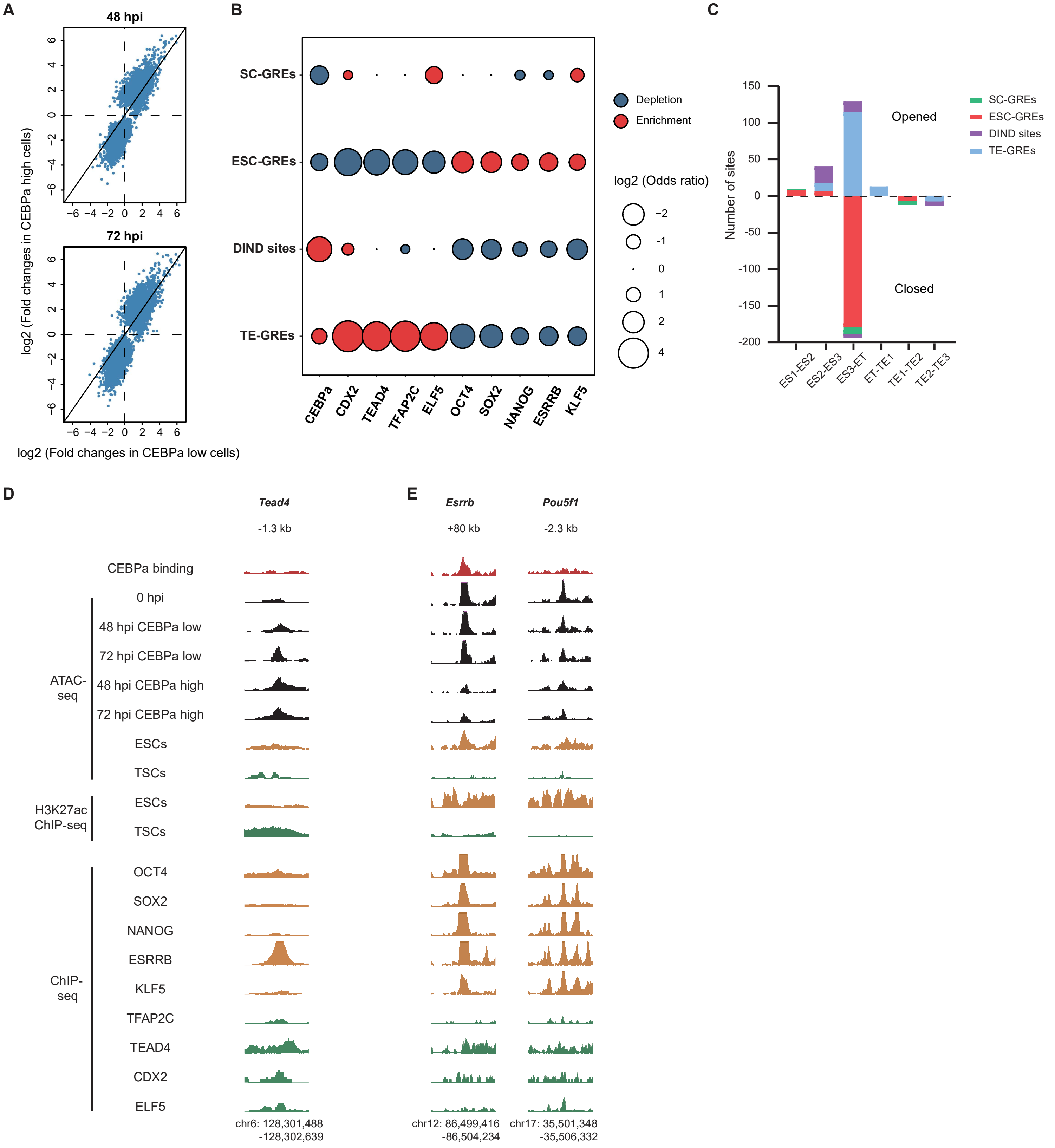
A
48 hpi
72 hpi
log2 (Fold changes in CEBPa high cells)
log2 (Fold changes in CEBPa low cells)
B
SC-GREs
ESC-GREs
DIND sites
TE-GREs
CEBPa
CDX2
TEAD4
TFAP2C
ELF5
OCT4
SOX2
NANOG
ESRRB
KLF5
Depletion
Enrichment
log2 (Odds ratio)
C
Number of sites
Opened
Closed
SC-GREs
ESC-GREs
DIND sites
TE-GREs
ES1-ES2
ES2-ES3
ES3-ET
ET-TE1
TE1-TE2
TE2-TE3
D
Tead4
-1.3 kb
CEBPa binding
0 hpi
48 hpi CEBPa low
72 hpi CEBPa low
48 hpi CEBPa high
72 hpi CEBPa high
ESCs
TSCs
ATAC-seq
H3K27ac ChIP-seq
ESCs
TSCs
ChIP-seq
OCT4
SOX2
NANOG
ESRRB
KLF5
TFAP2C
TEAD4
CDX2
ELF5
chr6: 128,301,488 -128,302,639
E
Esrrb
+80 kb
Pou5f1
-2.3 kb
chr12: 86,499,416 -86,504,234
chr17: 35,501,348 -35,506,332

Fig. S7. CEBPa overexpression enables the identification of TE- and ESC-associated GREs

(**A**) Chromatin accessibility of CEBPa^{low} and CEBPa^{high} cells at 48 and 72 hpi relative to uninduced cells.

(**B**) Binding enrichment/depletion of the factors shown in Fig. 7B analyzed by Fisher's exact test to score the odds ratio.

(**C**) Distribution of DAR clusters in opened and closed regions during cell state transitions as determined by single-cell analysis.

(**D** and **E**) Screenshots from the UCSC browser of individual TE-TF (D) and ESC-TF (E) enhancers, showing CEBPa binding at 24 hpi (red peaks) and time-resolved chromatin accessibility changes in CEBPa^{low} and CEBPa^{high} cells (peaks in black). They also show chromatin accessibility, H3K27ac decoration (*85, 86*), and binding of TE- and ESC-affiliated TFs in ESCs (brown peaks) and TSCs (green peaks) from published datasets (*35, 82-85*). The browser gains for the respective tracks were maintained constant in all panels shown.

A

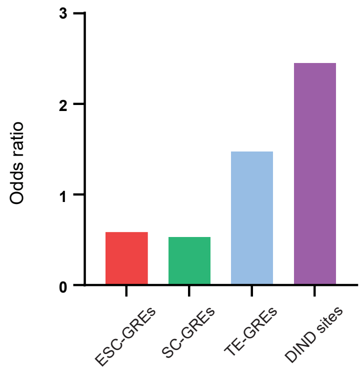

B

Activated TE-GREs

	Gata3 +60 kb	*Gata3* +58 kb	*Gata3* +18.1 kb	*Tead4* +21 kb	*Tead4* -1.3 kb	*Cdx2* -109 kb
OE: CEBPa bind.	Positive	Positive	Positive	(Positive)	(Positive)	Negative
OE: chrom. acc.	Opened	Opened	Opened	Opened	Opened	(Opened)
TE/ESC-TF bind.	TE	TE	TE	TE	TE	TE
Chromatin acc.	8C	4C, 8C	4C, 8C	4C, 8C	(4C), 8C	8C
H3K27ac decor.	4C, 8C	4C, 8C	4C	4C, 8C	4C, 8C	4C, 8C
	chr2:9,817,802 -9,819,110	chr2:9,820,468 -9,821,591	chr2:9,860,575 -9,861,993	chr6:128,278,616 -128,279,712	chr6:128,301,488 -128,302,639	chr5:147,416,396 -147,417,353

Primed TE-GREs

	Eomes -80 kb	*Elf5* +12 kb	*Id2* -13 kb
OE: CEBPa bind.	Positive	Positive	(Positive)
OE: chrom. acc.	Opened	Opened	Opened
TE/ESC-TF bind.	TE	TE	TE
Chromatin acc.	8C	8C	8C
H3K27ac decor.	Negative	Negative	Negative
	chr9:118,397,363 -118,398,643	chr2:103,435,557 -103,436,755	chr12:25,108,293 -25,109,777

DIND sites

	Gata3 +138 kb
OE: CEBPa bind.	Positive
OE: chrom. acc.	Opened
TE/ESC-TF bind.	Negative
Chromatin acc.	8C
H3K27Ac decor.	Negative
	chr2:9,739,166 -9,740,560

Standby TE-GREs

	Tfap2c -47 kb	*Eomes* -57 kb	*Eomes* -33 kb	*Id2* -19 kb	*Id2* -49 kb	*Id2* -74 kb	*Cdx2* +147 kb
OE: CEBPa bind.	Positive	Positive	Positive	Negative	Negative	(Positive)	Positive
OE: chrom. acc.	Opened	Opened	Opened	Opened	Opened	Opened	Opened
TE/ESC-TF bind.	TE	TE	TE	TE	TE	TE	TE
Chromatin acc.	Negative	Negative	Negative	Negative	Negative	Negative	Negative
H3K27ac decor.	Negative	Negative	Negative	Negative	Negative	Negative	Negative
	chr2:172,502,006 -172,502,768	chr9:118,420,675 -118,421,870	chr9:118,444,442 -118,445,343	chr12:25,114,228 -25,115,389	chr12:25,144,251 -25,145,411	chr12:25,169,018 -25,170,403	chr5:147,158,880 -147,159,834

C

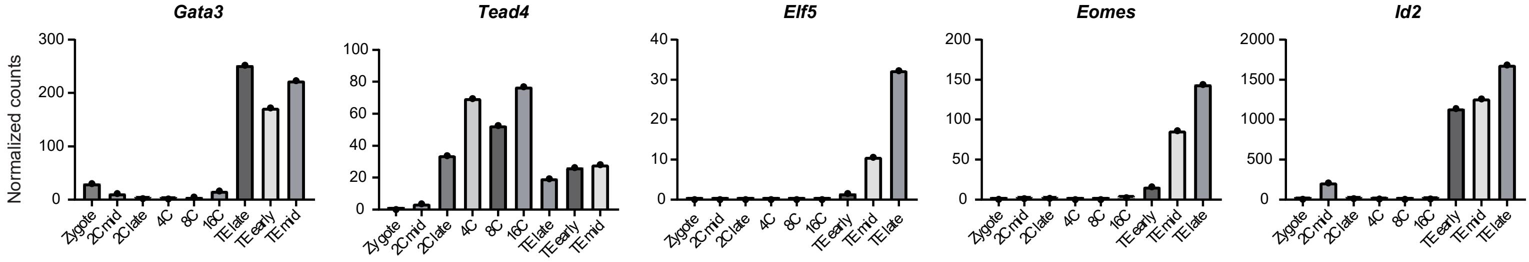

D

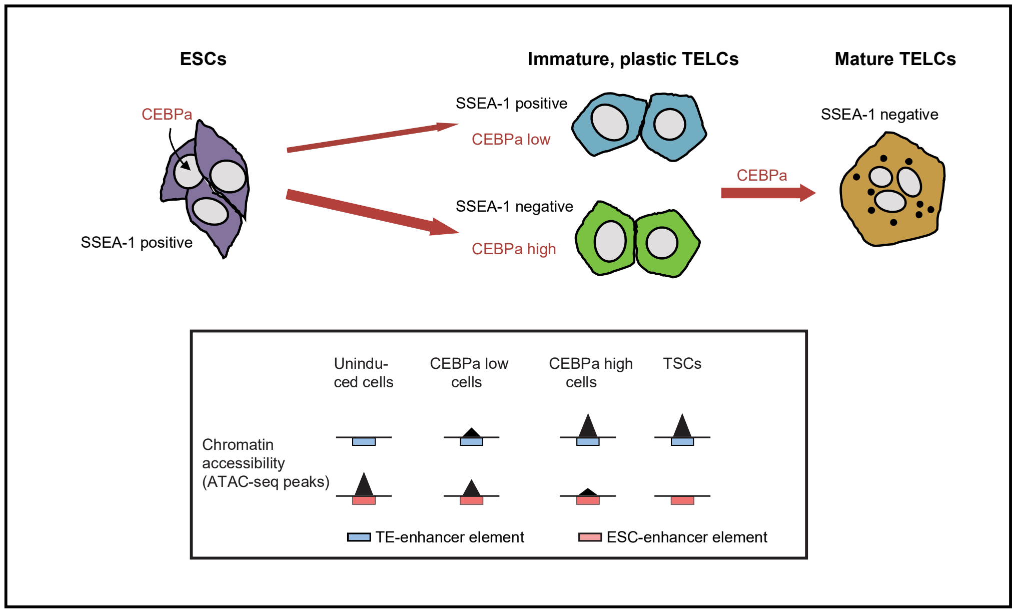

Fig. S8. TE-GREs of key trophectoderm transcription factors are accessible in early embryos

(**A**) Enrichment among the four groups within CEBPa regulome for sites showing chromatin accessibility in 4- to 8-cell embryos.

(**B**) Properties of regulatory sites detected in CEBPa-induced ESCs and in embryos. OE: CEBPa bind., indicates CEBPa binding sites at 24 hpi; OE: chrom. acc., chromatin accessibility changes after CEBPa induction; TE/ESC-TF bind., sites preferentially bound by TE- or ESC-associated TFs in TSCs and ESCs, respectively; Chromatin acc. and H3K27ac decor.: chromatin accessibility changes and H3K27ac decoration in early embryos. Weak signals are indicated with brackets. Fields highlighted in green: accessible/activated; in brown: not accessible/inactive.

(**C**) Gene expression of TE-associated TFs with activated and primed enhancers during preimplantation mouse embryo (*124*).

(**D**) Summary diagram of findings after activation of CEBPa in ESCs. Treatment with 4-OHT of ESC cells expressing CEBPa-ER induces nuclear expression of CEBPa and the formation of TELCs. Low and high levels of CEBPa expression result in slow (SSEA-1 positive) and fast (SSEA-1 negative) differentiating cells respectively, initiated by the dose-dependent opening of TE-affiliated enhancers and closing of ESC-affiliated enhancers. Insert depicts predicted chromatin accessibility changes at TE- and ESC- associated GREs.

Table. S1. List of housekeeping, cell cycle, and ribosomal genes excluded from the single-cell RNA-seq analysis

REFERENCES AND NOTES

1. C. H. Waddington, *Organisers and Genes* (Cambridge Univ. Press, 1940).

2. P. Bossard, K. S. Zaret, GATA transcription factors as potentiators of gut endoderm differentiation. *Development* **125**, 4909–4917 (1998).

3. K. Zaret, Developmental competence of the gut endoderm: Genetic potentiation by GATA and HNF3/fork head proteins. *Dev. Biol.* **209**, 1–10 (1999).

4. K. S. Zaret, Genetic programming of liver and pancreas progenitors: Lessons for stem-cell differentiation. *Nat. Rev. Genet.* **9**, 329–340 (2008).

5. A. Zalc, R. Sinha, G. S. Gulati, D. J. Wesche, P. Daszczuk, T. Swigut, I. L. Weissman, J. Wysocka, Reactivation of the pluripotency program precedes formation of the cranial neural crest. *Science* **371**, eabb4776 (2021).

6. M. Rostovskaya, G. G. Stirparo, A. Smith, Capacitation of human naïve pluripotent stem cells for multi-lineage differentiation. *Development* **146**, dev172916 (2019).

7. K. S. Zaret, J. S. Carroll, Pioneer transcription factors: Establishing competence for gene expression. *Genes Dev.* **25**, 2227–2241 (2011).

8. L. Li, F. Lai, L. Liu, X. Lu, X. Hu, B. Liu, Z. Lin, Q. Fan, F. Kong, Q. Xu, W. Xie, Lineage regulators TFAP2C and NR5A2 function as bipotency activators in totipotent embryos. *Nat. Struct. Mol. Biol.* **31**, 950–963 (2024).

9. M. Zhu, M. Meglicki, A. Lamba, P. Wang, C. Royer, K. Turner, M. A. Jauhar, C. Jones, T. Child, K. Coward, J. Na, M. Zernicka-Goetz, Tead4 and Tfap2c generate bipotency and a bistable switch in totipotent embryos to promote robust lineage diversification. *Nat. Struct. Mol. Biol.* **31**, 964–976 (2024).

10. M. D. White, J. Zenker, S. Bissiere, N. Plachta, Instructions for assembling the early mammalian embryo. *Dev. Cell* **45**, 667–679 (2018).

11. E. Posfai, J. P. Schell, A. Janiszewski, I. Rovic, A. Murray, B. Bradshaw, T. Yamakawa, T. Pardon, M. El Bakkali, I. Talon, N. De Geest, P. Kumar, S. K. To, S. Petropoulos, A. Jurisicova, V. Pasque, F. Lanner, J. Rossant, Evaluating totipotency using criteria of increasing stringency. *Nat. Cell Biol.* **23**, 49–60 (2021).

12. D. Jukam, S. A. M. Shariati, J. M. Skotheim, Zygotic genome activation in vertebrates. *Dev. Cell* **42**, 316–332 (2017).

13. F. Aoki, Zygotic gene activation in mice: Profile and regulation. *J. Reprod. Dev.* **68**, 79–84 (2022).

14. M. Zhu, M. Zernicka-Goetz, Principles of self-organization of the mammalian embryo. *Cell* **183**, 1467–1478 (2020).

15. M. Zernicka-Goetz, S. A. Morris, A. W. Bruce, Making a firm decision: Multifaceted regulation of cell fate in the early mouse embryo. *Nat. Rev. Genet.* **10**, 467–477 (2009).

16. K. Cockburn, J. Rossant, Making the blastocyst: Lessons from the mouse. *J. Clin. Invest.* **120**, 995–1003 (2010).

17. M. Zernicka-Goetz, Patterning of the embryo: The first spatial decisions in the life of a mouse. *Development* **129**, 815–829 (2002).

18. J. Rossant, P. P. Tam, Blastocyst lineage formation, early embryonic asymmetries and axis patterning in the mouse. *Development* **136**, 701–713 (2009).

19. N. Christodoulou, A. Weberling, D. Strathdee, K. I. Anderson, P. Timpson, M. Zernicka-Goetz, Morphogenesis of extra-embryonic tissues directs the remodelling of the mouse embryo at implantation. *Nat. Commun.* **10**, 3557 (2019).

20. M. Hemberger, C. W. Hanna, W. Dean, Mechanisms of early placental development in mouse and humans. *Nat. Rev. Genet.* **21**, 27–43 (2020).

21. K. Piotrowska, F. Wianny, R. A. Pedersen, M. Zernicka-Goetz, Blastomeres arising from the first cleavage division have distinguishable fates in normal mouse development. *Development* **128**, 3739–3748 (2001).

22. E. Casser, S. Israel, A. Witten, K. Schulte, S. Schlatt, V. Nordhoff, M. Boiani, Totipotency segregates between the sister blastomeres of two-cell stage mouse embryos. *Sci. Rep.* **7**, 8299 (2017).

23. S. Junyent, M. Meglicki, R. Vetter, R. Mandelbaum, C. King, E. M. Patel, L. Iwamoto-Stohl, C. Reynell, D. Y. Chen, P. Rubino, N. Arrach, R. J. Paulson, D. Iber, M. Zernicka-Goetz, The first two blastomeres contribute unequally to the human embryo. *Cell* **187**, 2838–2854.e17 (2024).

24. K. Piotrowska-Nitsche, M. Zernicka-Goetz, Spatial arrangement of individual 4-cell stage blastomeres and the order in which they are generated correlate with blastocyst pattern in the mouse embryo. *Mech. Dev.* **122**, 487–500 (2005).

25. M. E. Torres-Padilla, D. E. Parfitt, T. Kouzarides, M. Zernicka-Goetz, Histone arginine methylation regulates pluripotency in the early mouse embryo. *Nature* **445**, 214–218 (2007).

26. A. Burton, J. Muller, S. Tu, P. Padilla-Longoria, E. Guccione, M. E. Torres-Padilla, Single-cell profiling of epigenetic modifiers identifies PRDM14 as an inducer of cell fate in the mammalian embryo. *Cell Rep.* **5**, 687–701 (2013).

27. M. Goolam, A. Scialdone, S. J. L. Graham, I. C. Macaulay, A. Jedrusik, A. Hupalowska, T. Voet, J. C. Marioni, M. Zernicka-Goetz, Heterogeneity in Oct4 and Sox2 targets biases cell fate in 4-cell mouse embryos. *Cell* **165**, 61–74 (2016).

28. I. Tabansky, A. Lenarcic, R. W. Draft, K. Loulier, D. B. Keskin, J. Rosains, J. Rivera-Feliciano, J. W. Lichtman, J. Livet, J. N. Stern, J. R. Sanes, K. Eggan, Developmental bias in cleavage-stage mouse blastomeres. *Curr. Biol.* **23**, 21–31 (2013).

29. A. Hupalowska, A. Jedrusik, M. Zhu, M. T. Bedford, D. M. Glover, M. Zernicka-Goetz, CARM1 and paraspeckles regulate pre-implantation mouse embryo development. *Cell* **175**, 1902–1916.e13 (2018).

30. K. Krawczyk, E. Kosyl, K. Częścik-Łysyszyn, T. Wyszomirski, M. Maleszewski, Developmental capacity is unevenly distributed among single blastomeres of 2-cell and 4-cell stage mouse embryos. *Sci. Rep.* **11**, 21422 (2021).

31. M. Bischoff, D. E. Parfitt, M. Zernicka-Goetz, Formation of the embryonic-abembryonic axis of the mouse blastocyst: Relationships between orientation of early cleavage divisions and pattern of symmetric/asymmetric divisions. *Development* **135**, 953–962 (2008).

32. R. L. Gardner, Specification of embryonic axes begins before cleavage in normal mouse development. *Development* **128**, 839–847 (2001).

33. K. Piotrowska, M. Zernicka-Goetz, Role for sperm in spatial patterning of the early mouse embryo. *Nature* **409**, 517–521 (2001).

34. P. Strnad, S. Gunther, J. Reichmann, U. Krzic, B. Balazs, G. de Medeiros, N. Norlin, T. Hiiragi, L. Hufnagel, J. Ellenberg, Inverted light-sheet microscope for imaging mouse pre-implantation development. *Nat. Methods* **13**, 139–142 (2016).

35. M. Kinisu, Y. J. Choi, C. Cattoglio, K. Liu, H. Roux de Bezieux, R. Valbuena, N. Pum, S. Dudoit, H. Huang, Z. Xuan, S. Y. Kim, L. He, Klf5 establishes bi-potential cell fate by dual regulation of ICM and TE specification genes. *Cell Rep.* **37**, 109982 (2021).

36. J. Wu, B. Huang, H. Chen, Q. Yin, Y. Liu, Y. Xiang, B. Zhang, B. Liu, Q. Wang, W. Xia, W. Li, Y. Li, J. Ma, X. Peng, H. Zheng, J. Ming, W. Zhang, J. Zhang, G. Tian, F. Xu, Z. Chang, J. Na, X. Yang, W. Xie, The landscape of accessible chromatin in mammalian preimplantation embryos. *Nature* **534**, 652–657 (2016).

37. C. Y. Leung, M. Zernicka-Goetz, Angiomotin prevents pluripotent lineage differentiation in mouse embryos via Hippo pathway-dependent and -independent mechanisms. *Nat. Commun.* **4**, 2251 (2013).

38. Z. Wu, K. L. Guan, Hippo signaling in embryogenesis and development. *Trends Biochem. Sci.* **46**, 51–63 (2021).

39. E. Wicklow, S. Blij, T. Frum, Y. Hirate, R. A. Lang, H. Sasaki, A. Ralston, HIPPO pathway members restrict SOX2 to the inner cell mass where it promotes ICM fates in the mouse blastocyst. *PLOS Genet.* **10**, e1004618 (2014).

40. T. Frum, T. M. Murphy, A. Ralston, HIPPO signaling resolves embryonic cell fate conflicts during establishment of pluripotency in vivo. *eLife* **7**, e42298 (2018).

41. N. Nishioka, K. Inoue, K. Adachi, H. Kiyonari, M. Ota, A. Ralston, N. Yabuta, S. Hirahara, R. O. Stephenson, N. Ogonuki, R. Makita, H. Kurihara, E. M. Morin-Kensicki, H. Nojima, J. Rossant, K. Nakao, H. Niwa, H. Sasaki, The Hippo signaling pathway components Lats and Yap pattern Tead4 activity to distinguish mouse trophectoderm from inner cell mass. *Dev. Cell* **16**, 398–410 (2009).

42. A. Ralston, B. J. Cox, N. Nishioka, H. Sasaki, E. Chea, P. Rugg-Gunn, G. Guo, P. Robson, J. S. Draper, J. Rossant, Gata3 regulates trophoblast development downstream of Tead4 and in parallel to Cdx2. *Development* **137**, 395–403 (2010).

43. D. Strumpf, C. A. Mao, Y. Yamanaka, A. Ralston, K. Chawengsaksophak, F. Beck, J. Rossant, Cdx2 is required for correct cell fate specification and differentiation of trophectoderm in the mouse blastocyst. *Development* **132**, 2093–2102 (2005).

44. D. Huang, G. Guo, P. Yuan, A. Ralston, L. Sun, M. Huss, T. Mistri, L. Pinello, H. H. Ng, G. Yuan, J. Ji, J. Rossant, P. Robson, X. Han, The role of Cdx2 as a lineage specific transcriptional repressor for pluripotent network during the first developmental cell lineage segregation. *Sci. Rep.* **7**, 17156 (2017).

45. C. Chazaud, Y. Yamanaka, Lineage specification in the mouse preimplantation embryo. *Development* **143**, 1063–1074 (2016).

46. M. D. White, N. Plachta, Specification of the first mammalian cell lineages in vivo and in vitro. *Cold Spring Harb. Perspect. Biol.* **12**, a035634 (2020).

47. H. Niwa, Y. Toyooka, D. Shimosato, D. Strumpf, K. Takahashi, R. Yagi, J. Rossant, Interaction between Oct3/4 and Cdx2 determines trophectoderm differentiation. *Cell* **123**, 917–929 (2005).

48. F. Cambuli, A. Murray, W. Dean, D. Dudzinska, F. Krueger, S. Andrews, C. E. Senner, S. J. Cook, M. Hemberger, Epigenetic memory of the first cell fate decision prevents complete ES cell reprogramming into trophoblast. *Nat. Commun.* **5**, 5538 (2014).

49. W. H. Landschulz, P. F. Johnson, S. L. McKnight, The DNA binding domain of the rat liver nuclear protein C/EBP is bipartite. *Science* **243**, 1681–1688 (1989).

50. V. Heath, H. C. Suh, M. Holman, K. Renn, J. M. Gooya, S. Parkin, K. D. Klarmann, M. Ortiz, P. Johnson, J. Keller, C/EBPα deficiency results in hyperproliferation of hematopoietic progenitor cells and disrupts macrophage development in vitro and in vivo. *Blood* **104**, 1639–1647 (2004).

51. P. Zhang, J. Iwasaki-Arai, H. Iwasaki, M. L. Fenyus, T. Dayaram, B. M. Owens, H. Shigematsu, E. Levantini, C. S. Huettner, J. A. Lekstrom-Himes, K. Akashi, D. G. Tenen, Enhancement of hematopoietic stem cell repopulating capacity and self-renewal in the absence of the transcription factor C/EBPα. *Immunity* **21**, 853–863 (2004).

52. B. T. Porse, T. A. Pedersen, X. Xu, B. Lindberg, U. M. Wewer, L. Friis-Hansen, C. Nerlov, E2F repression by C/EBPα is required for adipogenesis and granulopoiesis in vivo. *Cell* **107**, 247–258 (2001).

53. P. Flodby, C. Barlow, H. Kylefjord, L. Ahrlund-Richter, K. G. Xanthopoulos, Increased hepatic cell proliferation and lung abnormalities in mice deficient in CCAAT/enhancer binding protein α. *J. Biol. Chem.* **271**, 24753–24760 (1996).

54. S. Pundhir, F. K. Bratt Lauridsen, M. B. Schuster, J. S. Jakobsen, Y. Ge, E. M. Schoof, N. Rapin, J. Waage, M. S. Hasemann, B. T. Porse, Enhancer and transcription factor dynamics during myeloid differentiation reveal an early differentiation block in Cebpa null progenitors. *Cell Rep.* **23**, 2744–2757 (2018).

55. M. Plana-Carmona, G. Stik, R. Bulteau, C. Segura-Morales, N. Alcazar, C. D. R. Wyatt, A. Klonizakis, L. de Andres-Aguayo, M. Gasnier, T. V. Tian, G. Torcal Garcia, M. Vila-Casadesus,

N. Plachta, M. Serrano, M. Francesconi, T. Graf, The trophectoderm acts as a niche for the inner cell mass through C/EBPα-regulated IL-6 signaling. *Stem Cell Reports* **17**, 1991–2004 (2022).

56. V. Begay, J. Smink, A. Leutz, Essential requirement of CCAAT/enhancer binding proteins in embryogenesis. *Mol. Cell. Biol.* **24**, 9744–9751 (2004).

57. H. Xie, M. Ye, R. Feng, T. Graf, Stepwise reprogramming of B cells into macrophages. *Cell* **117**, 663–676 (2004).

58. L. H. Bussmann, A. Schubert, T. P. Vu Manh, L. De Andres, S. C. Desbordes, M. Parra, T. Zimmermann, F. Rapino, J. Rodriguez-Ubreva, E. Ballestar, T. Graf, A robust and highly efficient immune cell reprogramming system. *Cell Stem Cell* **5**, 554–566 (2009).

59. F. Rapino, E. F. Robles, J. A. Richter-Larrea, E. M. Kallin, J. A. Martinez-Climent, T. Graf, C/EBPα induces highly efficient macrophage transdifferentiation of B lymphoma and leukemia cell lines and impairs their tumorigenicity. *Cell Rep.* **3**, 1153–1163 (2013).

60. C. van Oevelen, S. Collombet, G. Vicent, M. Hoogenkamp, C. Lepoivre, A. Badeaux, L. Bussmann, J. L. Sardina, D. Thieffry, M. Beato, Y. Shi, C. Bonifer, T. Graf, C/EBPα activates pre-existing and de novo macrophage enhancers during induced pre-B cell transdifferentiation and myelopoiesis. *Stem Cell Reports* **5**, 232–247 (2015).

61. B. Di Stefano, J. L. Sardina, C. van Oevelen, S. Collombet, E. M. Kallin, G. P. Vicent, J. Lu, D. Thieffry, M. Beato, T. Graf, C/EBPα poises B cells for rapid reprogramming into induced pluripotent stem cells. *Nature* **506**, 235–239 (2014).

62. G. Guo, M. Huss, G. Q. Tong, C. Wang, L. Li Sun, N. D. Clarke, P. Robson, Resolution of cell fate decisions revealed by single-cell gene expression analysis from zygote to blastocyst. *Dev. Cell* **18**, 675–685 (2010).

63. S. Petropoulos, D. Edsgard, B. Reinius, Q. Deng, S. P. Panula, S. Codeluppi, A. P. Reyes, S. Linnarsson, R. Sandberg, F. Lanner, Single-Cell RNA-Seq reveals lineage and X chromosome dynamics in human preimplantation embryos. *Cell* **167**, 285 (2016).

64. A. De Iaco, E. Planet, A. Coluccio, S. Verp, J. Duc, D. Trono, DUX-family transcription factors regulate zygotic genome activation in placental mammals. *Nat. Genet.* **49**, 941–945 (2017).

65. K. Sugie, S. Funaya, M. Kawamura, T. Nakamura, M. G. Suzuki, F. Aoki, Expression of Dux family genes in early preimplantation embryos. *Sci. Rep.* **10**, 19396 (2020).

66. N. Festuccia, S. Vandormael-Pournin, A. Chervova, A. Geiselmann, F. Langa-Vives, R.-X. Coux, I. Gonzalez, G. G. Collet, M. Cohen-Tannoudji, P. Navarro, Nr5a2 is dispensable for zygotic genome activation but essential for morula development. *Science* **386**, eadg7325 (2024).

67. F. Lai, L. Li, X. Hu, B. Liu, Z. Zhu, L. Liu, Q. Fan, H. Tian, K. Xu, X. Lu, Q. Li, K. Feng, L. Wang, Z. Lin, H. Deng, J. Li, W. Xie, NR5A2 connects zygotic genome activation to the first lineage segregation in totipotent embryos. *Cell Res.* **33**, 952–966 (2023).

68. J. Gassler, W. Kobayashi, I. Gáspár, S. Ruangroengkulrith, A. Mohanan, L. Gómez Hernández, P. Kravchenko, M. Kümmecke, A. Lalic, N. Rifel, R. J. Ashburn, M. Zaczek, A. Vallot, L. Cuenca Rico, S. Ladstätter, K. Tachibana, Zygotic genome activation by the totipotency pioneer factor Nr5a2. *Science* **378**, 1305–1315 (2022).

69. P. G. Hendrickson, J. A. Doráis, E. J. Grow, J. L. Whiddon, J.-W. Lim, C. L. Wike, B. D. Weaver, C. Pflueger, B. R. Emery, A. L. Wilcox, D. A. Nix, C. M. Peterson, S. J. Tapscott, D. T. Carrell, B. R. Cairns, Conserved roles of mouse DUX and human DUX4 in activating cleavage-stage genes and MERVL/HERVL retrotransposons. *Nat. Genet.* **49**, 925–934 (2017).

70. J. D. Buenrostro, B. Wu, H. Y. Chang, W. J. Greenleaf, ATAC-seq: A method for assaying chromatin accessibility genome-wide. *Curr. Protoc. Mol. Biol.* **109**, 21.29.21–21.29.29 (2015).

71. A. P. Russ, S. Wattler, W. H. Colledge, S. A. J. R. Aparicio, M. B. L. Carlton, J. J. Pearce, S. C. Barton, M. A. Surani, K. Ryan, M. C. Nehls, V. Wilson, M. J. Evans, Eomesodermin is required for mouse trophoblast development and mesoderm formation. *Nature* **404**, 95–99 (2000).

72. G. Shi, Y. Jin, Role of Oct4 in maintaining and regaining stem cell pluripotency. *Stem Cell Res. Ther.* **1**, 39 (2010).

73. D. G. Simmons, A. L. Fortier, J. C. Cross, Diverse subtypes and developmental origins of trophoblast giant cells in the mouse placenta. *Dev. Biol.* **304**, 567–578 (2007).

74. D. Solter, B. B. Knowles, Monoclonal antibody defining a stage-specific mouse embryonic antigen (SSEA-1). *Proc. Natl. Acad. Sci. U.S.A.* **75**, 5565–5569 (1978).

75. B. V. Natale, C. Schweitzer, M. Hughes, M. A. Globisch, R. Kotadia, E. Tremblay, P. Vu, J. C. Cross, D. R. C. Natale, Sca-1 identifies a trophoblast population with multipotent potential in the mid-gestation mouse placenta. *Sci. Rep.* **7**, 5575 (2017).

76. N. V. Varlakhanova, R. F. Cotterman, W. N. deVries, J. Morgan, L. R. Donahue, S. Murray, B. B. Knowles, P. S. Knoepfler, *myc* maintains embryonic stem cell pluripotency and self-renewal. *Differentiation* **80**, 9–19 (2010).

77. I. Korsunsky, N. Millard, J. Fan, K. Slowikowski, F. Zhang, K. Wei, Y. Baglaenko, M. Brenner, P. R. Loh, S. Raychaudhuri, Fast, sensitive and accurate integration of single-cell data with Harmony. *Nat. Methods* **16**, 1289–1296 (2019).

78. C. Alda-Catalinas, D. Bredikhin, I. Hernando-Herraez, F. Santos, O. Kubinyecz, M. A. Eckersley-Maslin, O. Stegle, W. Reik, A single-cell transcriptomics CRISPR-activation screen identifies epigenetic regulators of the zygotic genome activation program. *Cell Syst.* **11**, 25–41.e9 (2020).

79. I. Salvador-Martínez, M. Grillo, M. Averof, M. J. Telford, CeLaVi: An interactive cell lineage visualization tool. *Nucleic Acids Res.* **49**, W80–W85 (2021).

80. F. Yan, D. R. Powell, D. J. Curtis, N. C. Wong, From reads to insight: A hitchhiker's guide to ATAC-seq data analysis. *Genome Biol.* **21**, 22 (2020).

81. A. N. Schep, B. Wu, J. D. Buenrostro, W. J. Greenleaf, chromVAR: Inferring transcription-factor-associated accessibility from single-cell epigenomic data. *Nat. Methods* **14**, 975–978 (2017).

82. K. Adachi, I. Nikaido, H. Ohta, S. Ohtsuka, H. Ura, M. Kadota, T. Wakayama, H. R. Ueda, H. Niwa, Context-dependent wiring of Sox2 regulatory networks for self-renewal of embryonic and trophoblast stem cells. *Mol. Cell* **52**, 380–392 (2013).

83. P. Home, B. Saha, S. Ray, D. Dutta, S. Gunewardena, B. Yoo, A. Pal, J. L. Vivian, M. Larson, M. Petroff, P. G. Gallagher, V. P. Schulz, K. L. White, T. G. Golos, B. Behr, S. Paul, Altered subcellular localization of transcription factor TEAD4 regulates first mammalian cell lineage commitment. *Proc. Natl. Acad. Sci. U.S.A.* **109**, 7362–7367 (2012).

84. B. K. Lee, Y. J. Jang, M. Kim, L. LeBlanc, C. Rhee, J. Lee, S. Beck, W. Shen, J. Kim, Super-enhancer-guided mapping of regulatory networks controlling mouse trophoblast stem cells. *Nat. Commun.* **10**, 4749 (2019).

85. C. Chronis, P. Fiziev, B. Papp, S. Butz, G. Bonora, S. Sabri, J. Ernst, K. Plath, Cooperative binding of transcription factors orchestrates reprogramming. *Cell* **168**, 442–459.e20 (2017).

86. J. M. Frost, S. M. Amante, H. Okae, E. M. Jones, B. Ashley, R. M. Lewis, J. K. Cleal, M. P. Caley, T. Arima, T. Maffucci, M. R. Branco, Regulation of human trophoblast gene expression by endogenous retroviruses. *Nat. Struct. Mol. Biol.* **30**, 527–538 (2023).

87. A. Barral, K. S. Zaret, Pioneer factors: Roles and their regulation in development. *Trends Genet.* **40**, 134–148 (2024).

88. M. P. Creyghton, A. W. Cheng, G. G. Welstead, T. Kooistra, B. W. Carey, E. J. Steine, J. Hanna, M. A. Lodato, G. M. Frampton, P. A. Sharp, L. A. Boyer, R. A. Young, R. Jaenisch, Histone H3K27ac separates active from poised enhancers and predicts developmental state. *Proc. Natl. Acad. Sci.* **107**, 21931–21936 (2010).

89. R. Stadhouders, E. Vidal, F. Serra, B. Di Stefano, F. Le Dily, J. Quilez, A. Gomez, S. Collombet, C. Berenguer, Y. Cuartero, J. Hecht, G. J. Filion, M. Beato, M. A. Marti-Renom, T. Graf, Transcription factors orchestrate dynamic interplay between genome topology and gene regulation during cell reprogramming. *Nat. Genet.* **50**, 238–249 (2018).

90. H. Chen, M. Levo, L. Barinov, M. Fujioka, J. B. Jaynes, T. Gregor, Dynamic interplay between enhancer-promoter topology and gene activity. *Nat. Genet.* **50**, 1296–1303 (2018).

91. T. Pollex, A. Rabinowitz, M. C. Gambetta, R. Marco-Ferreres, R. R. Viales, A. Jankowski, C. Schaub, E. E. M. Furlong, Enhancer-promoter interactions become more instructive in the transition from cell-fate specification to tissue differentiation. *Nat. Genet.* **56**, 686–696 (2024).

92. B. de Laval, J. Maurizio, P. K. Kandalla, G. Brisou, L. Simonnet, C. Huber, G. Gimenez, O. Matcovitch-Natan, S. Reinhardt, E. David, A. Mildner, A. Leutz, B. Nadel, C. Bordi, I. Amit, S. Sarrazin, M. H. Sieweke, C/EBPβ-dependent epigenetic memory induces trained immunity in hematopoietic stem cells. *Cell Stem Cell* **26**, 657–674.e8 (2020).

93. Y. Yang, H. Zhou, X. Huang, C. Wu, K. Zheng, J. Deng, Y. Zheng, J. Wang, X. Chi, X. Ma, H. Pan, R. Shen, D. Pan, B. Liu, Innate immune and proinflammatory signals activate the Hippo pathway via a Tak1-STRIPAK-Tao axis. *Nat. Commun.* **15**, 145 (2024).

94. L. Heinke, Bookmarking pluripotency genes. *Nat. Rev. Mol. Cell Biol.* **25**, 160–160 (2024).

95. K. Piotrowska-Nitsche, A. Perea-Gomez, S. Haraguchi, M. Zernicka-Goetz, Four-cell stage mouse blastomeres have different developmental properties. *Development* **132**, 479–490 (2005).

96. M. D. White, J. F. Angiolini, Y. D. Alvarez, G. Kaur, Z. W. Zhao, E. Mocskos, L. Bruno, S. Bissiere, V. Levi, N. Plachta, Long-lived binding of Sox2 to DNA predicts cell fate in the four-cell mouse embryo. *Cell* **165**, 75–87 (2016).

97. C. S. Driscoll, J. Kim, J. G. Knott, The explosive discovery of TNT in early mouse embryos. *Nat. Struct. Mol. Biol.* **31**, 852–855 (2024).

98. H. Papuchova, P. A. Latos, Transcription factor networks in trophoblast development. *Cell. Mol. Life Sci.* **79**, 337 (2022).

99. N. Festuccia, N. Owens, P. Navarro, Esrrb, an estrogen-related receptor involved in early development, pluripotency, and reprogramming. *FEBS Lett.* **592**, 852–877 (2018).

100. H. Hosokawa, J. Ungerbäck, X. Wang, M. Matsumoto, K. I. Nakayama, S. M. Cohen, T. Tanaka, E. V. Rothenberg, Transcription factor PU.1 represses and activates gene expression in early T cells by redirecting partner transcription factor binding. *Immunity* **49**, 782 (2018).

101. G. Torcal Garcia, E. Kowenz-Leutz, T. V. Tian, A. Klonizakis, J. Lerner, L. De Andres-Aguayo, V. Sapozhnikova, C. Berenguer, M. P. Carmona, M. V. Casadesus, R. Bulteau, M. Francesconi, S. Peiro, P. Mertins, K. Zaret, A. Leutz, T. Graf, Carm1-arginine methylation of the transcription factor C/EBPα regulates transdifferentiation velocity. *eLife* **12**, e83951 (2023).

102. P. N. P. Singh, W. Gu, S. Madha, A. W. Lynch, P. Cejas, R. He, S. Bhattacharya, M. M. Gomez, M. G. Oser, M. Brown, H. W. Long, C. A. Meyer, Q. Zhou, R. A. Shivdasani, Transcription factor dynamics, oscillation, and functions in human enteroendocrine cell differentiation. bioRxiv 574746 [Preprint] (2024). https://doi.org/10.1101/2024.01.09.574746.

103. S. Yuan, J. Zhan, J. Zhang, Z. Liu, Z. Hou, C. Zhang, L. Yi, L. Gao, H. Zhao, Z.-J. Chen, J. Liu, K. Wu, Human zygotic genome activation is initiated from paternal genome. *Cell Discov.* **9**, 13 (2023).

104. Y. Nakatake, S. B. H. Ko, A. A. Sharov, S. Wakabayashi, M. Murakami, M. Sakota, N. Chikazawa, C. Ookura, S. Sato, N. Ito, M. Ishikawa-Hirayama, S. S. Mak, L. M. Jakt, T. Ueno, K. Hiratsuka, M. Matsushita, S. K. Goparaju, T. Akiyama, K. I. Ishiguro, M. Oda, N. Gouda, A. Umezawa, H. Akutsu, K. Nishimura, R. Matoba, O. Ohara, M. S. H. Ko, Generation and Profiling of 2,135 human ESC lines for the systematic analyses of cell states perturbed by inducing single transcription factors. *Cell Rep.* **31**, 107655 (2020).

105. L. A. Cirillo, K. S. Zaret, An early developmental transcription factor complex that is more stable on nucleosome core particles than on free DNA. *Mol. Cell* **4**, 961–969 (1999).

106. M. Zernicka-Goetz, J. Pines, S. McLean Hunter, J. P. Dixon, K. R. Siemering, J. Haseloff, M. J. Evans, Following cell fate in the living mouse embryo. *Development* **124**, 1133–1137 (1997).

107. G. Pau, F. Fuchs, O. Sklyar, M. Boutros, W. Huber, EBImage—An R package for image processing with applications to cellular phenotypes. *Bioinformatics* **26**, 979–981 (2010).

108. A. Dobin, C. A. Davis, F. Schlesinger, J. Drenkow, C. Zaleski, S. Jha, P. Batut, M. Chaisson, T. R. Gingeras, STAR: Ultrafast universal RNA-seq aligner. *Bioinformatics* **29**, 15–21 (2013).

109. M. I. Love, W. Huber, S. Anders, Moderated estimation of fold change and dispersion for RNA-seq data with DESeq2. *Genome Biol.* **15**, 550 (2014).

110. M. Martin, Cutadapt removes adapter sequences from high-throughput sequencing reads. *EMBnet J.* **17**, 10.14806/ej.17.1.200 (2011).

111. B. Langmead, S. L. Salzberg, Fast gapped-read alignment with Bowtie 2. *Nat. Methods* **9**, 357–359 (2012).

112. H. Li, B. Handsaker, A. Wysoker, T. Fennell, J. Ruan, N. Homer, G. Marth, G. Abecasis, R. Durbin, 1000 Genome Project Data Processing Subgroup, The sequence alignment/map format and SAMtools. *Bioinformatics* **25**, 2078–2079 (2009).

113. F. Ramírez, D. P. Ryan, B. Grüning, V. Bhardwaj, F. Kilpert, A. S. Richter, S. Heyne, F. Dündar, T. Manke, deepTools2: A next generation web server for deep-sequencing data analysis. *Nucleic Acids Res.* **44**, W160–W165 (2016).

114. Y. Zhang, T. Liu, C. A. Meyer, J. Eeckhoute, D. S. Johnson, B. E. Bernstein, C. Nusbaum, R. M. Myers, M. Brown, W. Li, X. S. Liu, Model-based analysis of ChIP-Seq (MACS). *Genome Biol.* **9**, R137 (2008).

115. T. L. Bailey, J. Johnson, C. E. Grant, W. S. Noble, The MEME suite. *Nucleic Acids Res.* **43**, W39–W49 (2015).

116. C. S. Ross-Innes, R. Stark, A. E. Teschendorff, K. A. Holmes, H. R. Ali, M. J. Dunning, G. D. Brown, O. Gojis, I. O. Ellis, A. R. Green, S. Ali, S.-F. Chin, C. Palmieri, C. Caldas, J. S. Carroll, Differential oestrogen receptor binding is associated with clinical outcome in breast cancer. *Nature* **481**, 389–393 (2012).

117. Y. Hao, S. Hao, E. Andersen-Nissen, W. M. Mauck, III, S. Zheng, A. Butler, M. J. Lee, A. J. Wilk, C. Darby, M. Zager, P. Hoffman, M. Stoeckius, E. Papalexi, E. P. Mimitou, J. Jain, A.

Srivastava, T. Stuart, L. M. Fleming, B. Yeung, A. J. Rogers, J. M. McElrath, C. A. Blish, R. Gottardo, P. Smibert, R. Satija, Integrated analysis of multimodal single-cell data. *Cell* **184**, 3573–3587.e29 (2021).

118. T. Stuart, A. Srivastava, S. Madad, C. A. Lareau, R. Satija, Single-cell chromatin state analysis with Signac. *Nat. Methods* **18**, 1333–1341 (2021).

119. V. Bergen, M. Lange, S. Peidli, F. A. Wolf, F. J. Theis, Generalizing RNA velocity to transient cell states through dynamical modeling. *Nat. Biotechnol.* **38**, 1408–1414 (2020).

120. K. R. Moon, D. van Dijk, Z. Wang, S. Gigante, D. B. Burkhardt, W. S. Chen, K. Yim, A. van den Elzen, M. J. Hirn, R. R. Coifman, N. B. Ivanova, G. Wolf, S. Krishnaswamy, Visualizing structure and transitions in high-dimensional biological data. *Nat. Biotechnol.* **37**, 1482–1492 (2019).

121. Z. Gu, R. Eils, M. Schlesner, Complex heatmaps reveal patterns and correlations in multidimensional genomic data. *Bioinformatics* **32**, 2847–2849 (2016).

122. R. Stark, G. Brown, DiffBind: Differential binding analysis of ChIP-Seq peak data, R package version 100 (2011).

123. B. Liu, Y. He, X. Wu, Z. Lin, J. Ma, Y. Qiu, Y. Xiang, F. Kong, F. Lai, M. Pal, P. Wang, J. Ming, B. Zhang, Q. Wang, J. Wu, W. Xia, W. Shen, J. Na, M. E. Torres-Padilla, J. Li, W. Xie, Mapping putative enhancers in mouse oocytes and early embryos reveals TCF3/12 as key folliculogenesis regulators. *Nat. Cell Biol.* **26**, 962–974 (2024).

124. Q. Deng, D. Ramsköld, B. Reinius, R. Sandberg, Single-cell RNA-seq reveals dynamic, random monoallelic gene expression in mammalian cells. *Science* **343**, 193–196 (2014).
